# Supplementary material for: Fundamental nursing care focusing on older people’s needs and continuity of long-term care: a scoping review protocol
Source: BMJ Open. 2023 Mar 28;13(3):e069798. doi: 10.1136/bmjopen-2022-069798 (PMC10069605; doi:10.1136/bmjopen-2022-069798)
Supplement: Supplementary data [file bmjopen-2022-069798supp002.pdf]

**Additional file 2**

Search blocks PubMed – tested 09.03.2023

| Nr | Population - Older people | Mesh & All Fields |
|---|---|---|
| #1 | Aged [MT] | 3,438,107 |
| #2 | Elderly [MT] | 3,438,107 |
| #3 | Older adult* [AF] | 287,242 |
| #4 | Old [AF] | 1,256,543 |
| #5 | Older people [AF] | 432,325 |
| #6 | Senior* [AF] | 91,963 |
| | | |
| #7 | ((((((#1) OR (#2)) OR (#3)) OR (#4)) OR (#5)) OR (#6) | 4,706,859 |
| | | |

| Nr | Population - Nursing staff | Mesh & All Fields |
|---|---|---|
| #8 | Allied health professional* [MT] | 5,371 |
| #9 | Licenced practical nurse [MT] | 936 |
| #10 | Nurse*[MT] | 161,299 |
| #11 | Registered nurse* [AF] | 21,175 |
| #12 | Licensed practice nurse* [AF] | 3,094 |
| #13 | Registered practical nurse* [AF] | 10,147 |
| #14 | Licensed vocational nurse* [AF] | 338 |
| #15 | Nurse assistant* [AF] | 16,897 |
| #16 | Health care assistant [AF] | 72,382 |
| #17 | Formal caregiver [AF] | 3,446 |
| #18 | Health care professional [AF] | 811,662 |
| #19 | Practicing nurse* [AF] | 125,708 |
| #20 | Professional nurs* [AF] | 124,771 |
| #21 | Associate professional nurs* [AF] | 22,698 |
| #22 | Nursing aide* [AF] | 3,199 |
| #23 | Nursing assistant* [AF] | 19,454 |
| #24 | Nurse specialist* [AF] | 17,255 |
| | | |
| #25 | ((((((((((((#8) OR (#9) OR (#10) OR (#10)) OR (#11)) OR (#12)) OR (#13)) OR (#14) OR (#15)) OR (#16) OR (#17) OR (#18)) OR (#19)) OR (#20) OR (#21)) OR (#22) OR (#23) | 977,683 |
| | | |

| Nr | Phenomenon of Interest - Continuity of care | Mesh & All Fields |
|---|---|---|
| #26 | Continuity of patient care [MT] | 285,774 |
| #27 | Continuity of care [AF] | 297,752 |
| #28 | Continuity of nursing care [AF] | 70,997 |
| #29 | Care continuity [AF] | 297,752 |
| #30 | Informational continuity [AF] | 135,277 |
| #31 | Relational continuity [AF] | 257,556 |
| #32 | Interpersonal continuity [AF] | 5,836 |
| #33 | Longitudinal continuity [AF] | 28,001 |
| #34 | Management continuity [AF] | 250,481 |
| | | |
| #35 | ((((((((((#26)) OR (#27)) OR (#28)) OR (#29)) OR (#30)) OR (#31) OR (#32)) OR (#33) OR (#34)) | 803,082 |
| | | |

| Nr | Phenomenon of Interest - Care & nursing | Mesh & All Fields |
|---|---|---|
| #36 | Nursing care [MT] | 141,040 |
| #37 | Nursing [MT] | 262,763 |
| #38 | Patient* care need* [AF] | 364,406 |
| #39 | Basic care [AF] | 59,931 |
| #40 | Essence of care [AF] | 2,524 |
| #41 | Essential care [AF] | 89,592 |
| #42 | Fundamental care [AF] | 22,791 |
| #43 | Eldercare [AF] | 598 |
| #44 | Elderly care [AF] | 78,561 |
| #45 | Geriatric care [AF] | 71,595 |
| #46 | Care of older persons [AF] | 122,045 |

## Additional file 2

| | | |
|---|---|---|
| #47 | Care of elderly persons [AF] | 870,268 |
| #48 | Care of aged persons [AF] | 888,440 |
| #49 | Old age assistance [AF] | 2,261 |
| #50 | Care [AF] | 3,134,474 |
| #51 | Care need* [AF] | 592,274 |
| #52 | Physical care need* [AF] | 66,455 |
| #53 | Basic nursing need* [AF] | 4,453 |
| #54 | Physiological need* [AF] | 75,595 |
| #55 | Biological need* [AF] | 430,845 |
| #56 | Psychosocial need* [AF] | 35,371 |
| #57 | Physical care [AF] | 292,127 |
| #58 | Advanced care [AF] | 182,347 |
| #59 | Support [AF] | 11,411,024 |
| #60 | Relationship* [AF] | 2,552,156 |
| #61 | Caring network [AF] | 4,047 |
| #62 | Care plan [AF] | 64,506 |
| #63 | Compassionate care [AF] | 4,014 |
| #64 | Core nursing [AF] | 10,215 |
| #65 | ((((((((((((#36) OR (#37)) OR (#38) OR (#39) OR (#40) OR (#41) OR (#42)) OR (#43)) OR (#44)) OR (#45)) OR (#46) OR (#47)) OR (#48) OR (#49)) OR (#50) OR (#51) OR (#52)) OR (#53) OR (#54)) OR (#55) OR (#56) OR (#57) OR (#58)) OR (#59) OR (#60) OR (#61) OR (#62) OR (#63) OR (#64) | 15,059,100 |

| Nr | Phenomenon of Interest - Models, frameworks and interventions of care | Mesh & All Fields |
|---|---|---|
| #66 | Nursing model [MT] | 12,236 |
| #67 | Nursing process [MT] | 86,531 |
| #68 | Critical pathway [MT] | 7,710 |
| #69 | Clinical protocol [MT] | 188,274 |
| #70 | Practice guideline [MT] | 137,215 |
| #71 | Care model* [AF] | 449,813 |
| #72 | Nursing framework* [AF] | 24,500 |
| #73 | Care framework* [AF] | 74,236 |
| #74 | Nursing approach [AF] | 72,784 |
| #75 | Care approach [AF] | 328,634 |
| #76 | Nursing programme [AF] | 117,616 |
| #77 | Care programme [AF] | 438,548 |
| #78 | Nursing intervention* [AF] | 105,830 |
| #79 | Nurse-led [AF] | 4,926 |
| #80 | Patient care pathway [AF] | 27,988 |
| #81 | Patient pathway [AF] | 260,075 |
| #82 | Care pathway [AF] | 103,209 |
| #83 | Clinical pathway [AF] | 208,344 |
| #84 | Clinical recommendation* [AF] | 146,949 |
| #85 | Clinical path [AF] | 87,189 |
| #86 | Clinical care plan [AF] | 25,248 |
| #87 | Care path [AF] | 9,242 |
| #88 | Care guideline [AF] | 107,818 |
| #89 | Care recommendation* [AF] | 113,660 |
| #90 | Care protocol [AF] | 1,207 |
| #91 | (((((((((((((((((((((((((#66)) OR (#67) OR (#68)) OR (#69) OR (#70) OR (#71)) OR (#72) OR (#73)) OR (#74) OR (#75) OR (#76) OR (#77) OR (#78)) OR (#79) OR (#80) OR (#81) OR (#82) OR (#83) OR (#84)) OR (#85) OR (#86) OR (#87) OR (#88) OR (#89) OR (#90) | 2,061,156 |

| Nr | Context - Long term care, home healthcare, home nursing, and other care facilities | Mesh & All Fields |
|---|---|---|
| #92 | Community health services [MT] | 329,298 |
| #93 | Home health services [MT] | 125,852 |
| #94 | Home health nursing [MT] | 375 |
| #95 | Long term care [MT] | 28,240 |
| #96 | Primary health care [MT] | 188,798 |
| #97 | Nursing home* [MT] | 43,998 |
| #98 | Housing for the elderly [MT] | 1,652 |
| #99 | Housing [MT] | 36,444 |
| #100 | Primary care [AF] | 570,684 |
| #101 | Community care [AF] | 331,461 |

**Additional file 2**

| | | |
|---|---|---|
| #102 | Community health services [AF] | 438,391 |
| #103 | Municipal care [AF] | 60,864 |
| #104 | Home care [AF] | 961,037 |
| #105 | Home dwelling [AF] | 30,403 |
| #106 | Home healthcare [AF] | 74,095 |
| #107 | Home and community-based care [AF] | 5,249 |
| #108 | Home and community-based services [AF] | 4,519 |
| #109 | Longitudinal care [AF] | 62,908 |
| #110 | Assisted living [AF] | 30,390 |
| #111 | Independent living [AF] | 37,878 |
| #112 | Residential care aged facilities [AF] | 4,310 |
| #113 | Sheltered accommodation [AF] | 309 |
| #114 | Sheltered housing [AF] | 2,605 |
| #115 | Residential care [AF] | 16,366 |
| #116 | Care home [AF] | 161,305 |
| #117 | Day care [AF] | 174,595 |
| #118 | Special accommodation [AF] | 17,887 |
| | | |
| #119 | ((((((((((((((((((((((((((((((#92)) OR (#93)) OR (#94) OR (#95)) OR (#96) OR (#97)) OR (#98) OR (#99) OR (#100)) OR (#101) OR (#102)) OR (#103) OR (#104) OR (#105) OR (#106) OR (#107)) OR (#108) OR (#109) OR (#110) OR (#111) OR (#112) OR (#113)) OR (#114) OR (#115) OR (#116)) OR (#117) OR (#118) | 2,114,266 |
| | | |
