# Supplementary material for: Fundamental nursing care focusing on older people’s needs and continuity of long-term care: a scoping review protocol
Source: BMJ Open. 2023 Mar 28;13(3):e069798. doi: 10.1136/bmjopen-2022-069798 (PMC10069605; doi:10.1136/bmjopen-2022-069798)
Supplement: Author's manuscript [file bmjopen-2022-069798.draft_revisions.pdf]

BMJ Open

BMJ Open is committed to open peer review. As part of this commitment we make the peer review history of every article we publish publicly available.

When an article is published we post the peer reviewers' comments and the authors' responses online. We also post the versions of the paper that were used during peer review. These are the versions that the peer review comments apply to.

The versions of the paper that follow are the versions that were submitted during the peer review process. They are not the versions of record or the final published versions. They should not be cited or distributed as the published version of this manuscript.

BMJ Open is an open access journal and the full, final, typeset and author-corrected version of record of the manuscript is available on our site with no access controls, subscription charges or pay-per-view fees (http://bmjopen.bmj.com).

If you have any questions on BMJ Open's open peer review process please email info.bmjopen@bmj.com

BMJ Open

How Nursing Care and Models of Care Are Described in Relation to Older People's Basic Care Needs and Continuity of Long-term Care: A Scoping Review Protocol

Journal:	*BMJ Open*
Manuscript ID	bmjopen-2022-069798
Article Type:	Protocol
Date Submitted by the Author:	04-Nov-2022
Complete List of Authors:	Nordaunet, Ole Martin; Lovisenberg Diaconal University College,<br>Gjevjon, Edith; Lovisenberg Diaconal University College, Bachelor Education in Nursing<br>Olsson, Cecilia; Karlstad University<br>Aagaard , Hanne; Lovisenberg Diaconal University College<br>Borglin, Gunilla; Lovisenberg Diaconal University College, Department of Nursing
Keywords:	Quality in health care < HEALTH SERVICES ADMINISTRATION & MANAGEMENT, PRIMARY CARE, STATISTICS & RESEARCH METHODS

SCHOLARONE™
Manuscripts

How Nursing Care and Models of Care Are Described in Relation to Older People's Basic Care Needs and Continuity of Long-term Care: A Scoping Review Protocol

Nordaunet O.M., RN, MSc, PhD-student [1, 2] Gjevjon E. R., RN, MSc, PhD, Professor of nursing[1] Olsson C., RN, MSc, PhD, Associate Professor[2] Aagaard H., RN, MSc, PhD, Professor[1] Borglin G., Professor of Nursing[1]

[1]Lovisenberg Diaconal University College, Department of Bachelor Education (Nursing). Lovisenberggata 15B, NO-0456 Oslo, Norway

[2] Karlstad University, Institute of Health Sciences, Department of Nursing. Universitetsgatan 2, 651 88 Karlstad, Sweden

Ole Martin Nordaunet:	https://orcid.org/0000-0002-6061-0033
Edith Roth Gjevjon:	https://orcid.org/0000-0002-9656-522X
Cecilia Olsson:	https://orcid.org/0000-0002-0944-5650
Hanne Aagaard:	https://orcid.org/0000-0002-7810-9298
Gunilla Borglin:	https://orcid.org/0000-0002-7934-6949

Word count (incl. Two tables): 2871

Corresponding author:

Ole Martin Nordaunet

Lovisenberg Diaconal University College,

Lovisenberggata 15b, 0456 Oslo

Norway

E-mail: Ole.martin.nordaunet@ldh.no

Phone: + 47 997 313 70

For peer review only

ABSTRACT

Introduction: Nowadays, more older people than ever before receive long-term care in the community, and this group of older people is regularly portrayed as presenting comprehensive health care needs. The latter mainly refer to older people's physical, social, and psychosocial care needs, which are often described in nursing research as representing basic, essential, or fundamental care. However, research implies that at times, older people's basic care needs are poorly met in this context, reflecting fragmented care and a lack of continuity of nursing care. Therefore, this scoping review aims to map the literature on nursing care in relation to basic care, continuity of care and described nursing interventions targeting basic care among older people in long-term care.

Method and analysis: The upcoming scoping review will be conducted in accordance with Arksey and O'Malley's methodological framework, as well as with methodological updates by Levac et al. Peters et al. and Bradbury-Jones et al. Eligible literature will be searched for in the PubMed, CINAHL and PsychINFO databases. The results will be collated, summarised, and reported. This protocol adheres to the PRISMA protocol checklist.

Ethics and dissemination: This literature review will take into consideration ethical reporting in primary research as part of the quality assessment. Results of this scoping review will be submitted to an open-access peer-reviewed journal. This scoping review are part of an initial phase of developing a complex intervention which aims for increased quality of care in relation to basic care needs and continuity of care among older people in long-term care.

Registration: Open Science Framework https://doi.org/10.17605/OSF.IO/XJ39E

For peer review only

STRENGHTS AND LIMITATION OF THE STUDY

- Comprehensive development of search strategies to ensure optimal uptake of papers
- Utilising well established methodology for scoping reviews as well as incorporating methodological advancements
- Quality assessment of included papers likely to add further information
- Only peer-reviewed papers written in English and exclusion of grey literature might limit results

For peer review only

INTRODUCTION

The shift from hospital-based care to long-term care has resulted in older people (65 years and older) spending less time in hospital-based care and instead receiving more care in a long-term care context. This shift coincides with older people's preferences in relation to ageing in place or the wish to remain in their own homes for as long as possible.[1-4] This has resulted in increased demand and pressure on the health care services provided within long-term care contexts.[5, 6] Long-term care is defined by the National Institute on Aging as: *"A range of preventive and responsive care and supports, primarily for older adults, provided by either not-for-profit and for-profit providers, or unpaid caregivers in settings that are not location specific and thus include designated buildings, or in home and community-based settings"* (p. 14).[7] Research concerning older people in long-term care contexts, such as nursing homes, sheltered housing or home health care are often described as presenting with multiple chronic conditions, high age and requiring comprehensive basic care, related to nutrition, mobility, elimination, personal hygiene and so on.[8-10] Attending to these care needs is within the remit of nursing and is labelled in the literature as either basic, essential, or fundamental care.[11-15] Basic care needs typically depict fundamental human needs such as a safe environment, communication, breathing, eating and drinking, elimination, personal cleansing, dressing, rest, controlling body temperature, mobilising, working, playing, expressing sexuality, sleeping and end-of-life care.[12] However, research implies that the care targeting older people's basic, physiological, relational, and psychosocial needs in long-term care contexts is often fragmented, missed, deprioritised, rationed and poorly attended to.[16-22] Thus, unmet, or rationed care is not uncommon,[23 24] raising concerns about whether nursing staff can consistently ensure older people's basic care needs. Pressure on resources, austerity of long-term care services and a lack of competent health care personnel may aggravate and sustain fragmented long-term care, decreasing the quality of care in this context.[21, 25-27] One possible way to aid nursing staff in promoting basic care can be by implementing evidence-based practices supporting them in assessing, evaluating, and implementing basic care within long-term care contexts. As such, structured and logical models of care might be one part of a solution in ensuring optimal continuity of care and, thus, quality of care for older people in long-term care.

For peer review only

Recent literature reviews suggest that the focus on models of care concerning basic care and continuity of care among older people in long-term care is scant[28] and that models of care are sparsely implemented in long-term care contexts.[29] Models of care should be understood as a description of how to deliver care within the wider context of health care services,[30] here being the long-term care context. Nursing staff working under models of care are described as being more likely to work systematically and towards mutually set goals.[8] Working in accordance with models of care implies that nursing staff can assess and evaluate the delivery of older people's basic care on a regular basis; this could promote basic care in a predictable manner that corresponds to older patients' preferences and basic care needs continuously over time (i.e continuity of care). Research suggests that unnecessary hospitalisations among older people are rooted in poor continuity of care arising from health care services lacking the ability to synchronise the main components within continuity of care, namely informational, management and relational continuity.[31] Continuity of care is also described as a key indicator for quality of care, as well as essential in preventing the unnecessary use of specialist health care services, such as hospital admissions, lowering cost and decreasing mortality.[31-33] Consequently, models of care could be useful tools in preventing a poor empirical understanding of basic care needs among older people and in ensuring that their care needs are continuously met. Thus, our scoping review aims to map the literature on nursing care in relation to basic care and continuity of care while describing those nursing interventions targeting basic care (as models of care, patient care pathways or clinical practice guidelines) among older people in long-term care.

METHODS

Our scoping review will be based on Arksey and O'Malley's methodological framework[34] which is recognized by its iterative six-stage process, as well as updated methodology.[35-37] Scoping review methodology is particularly useful in assessing key characteristics and identifying knowledge gaps in the literature within complex topics.[34, 38, 39] Two authors (ERG & GB) will collaborate closely with the first author (OMN) in all the six stages, while two authors (HG & CO) will provide critical assessment and intellectual feedback within the overall process of stages one to six. This protocol is designed and reported in accordance with the preferred reporting items for the systematic review and meta-analysis protocols checklist, PRISMA-P (Supplemental material 1).[40] Our upcoming scoping review will adhere to the PRISMA-Scoping Review checklist[41] and is registered at Open Science Framework, last

For peer review only

updated the 22nd of March 2022 (https://doi.org/10.17605/OSF.IO/XJ39E). Operationalisation of core concepts are presented in box 1.

Box 1. Operationalisation of core concepts

Long-term care are here operationalised as:
- Health care delivered over prolonged periods of time in the patient's home, nursing home or sheltered housing, but can as well be delivered in other contexts in the community, such as, but not limited to day care centres, assisted living facilities and retirement homes.[7, 42-44]

Continuity of care are here operationalised as:
- *'The degree to which a series of discrete health care events is experienced by people as coherent and interconnected over time and consistent with their health needs and preferences'*.[45]

Key stakeholders are here operationalised as:
- Older people, their relatives, significant others, and nurses

Nurses and/or nursing staff are here operationalised as:
- Practicing nurses, professional nurses, associate professional nurses, healthcare assistants, nursing aides, nurse specialists, registered nurses, registered practical, licenced practice nurses, hereafter referred to as nurses.[46, 47]

For peer review only

Stage one: Identifying the research questions

Research questions in scoping reviews adopt a broad approach, even so the research questions need clarity, focus and direction.[39] To aid in the development of broad research questions, the population, phenomena of interest, context and study design (PICoS) framework by Methley et al. will be utilised (Table 1).[48]

Table 1. PICoS framework

Criteria	Determinants
Population	- Older people in need of care (>65) years<br>- Relatives in the broad understanding<br>- Nurses as defined in box 1.
(Phenomenon of) Interest	- Care needs<br>- Models of care, patient care pathways or clinical practice guidelines<br>- Continuity of care
Context	- Long-term care contexts, as operationalised in box 1
Study design	- All types of primary research designs

The scoping review will tentatively explore the following questions:

Question 1. Which basic nursing care needs are researched and presented among older people in long-term care, and how are they described by key stakeholders?

Question 2. What nursing interventions (as models of care, patient care pathways or clinical practice guidelines) are described as targeting older people's basic care needs and/or continuity of care in long-term care?

Additionally, sub-questions to map out what kind of research designs, contextual differences and what target audience research is aimed towards can be examined as well.

Stage two: Identifying relevant studies

In scoping reviews, a variety of sources are typically relevant to include to answer the research questions.[49] However, our main source of data is English-written, peer-reviewed, primary research from the Public/Publisher MEDLINE (PubMed), Cumulative Index to Nursing and Allied Health Literature (CINAHL) and Psychological Information Database (PsychINFO) databases. The rationale behind only referring to peer-reviewed literature assumes that this type of literature will provide the necessary foundation to answer the research questions. All authors have been part of the development of a comprehensive search strategy. The first author will consult an information specialist to refine the developed search strategy early in the piloting stages of the search strategy. The iterative approach of a scoping review encourages the researcher to strike a balance between comprehensiveness and manageability of search results.[37] Hence, the initial developed search strategy will be comprehensive, but as we gain experience through piloting searches our strategy will be narrowed down. Both medical subject headings (MeSH), keywords and Boolean operators make up the search strategy.[50] The planned filters used is 'age' (65 years and older), English language and publication range 2002-2022. The research team will examine the search strategies tailored for each database, and at a later stage an additional information specialist will peer review the search strategy. A preliminary search strategy for PubMed is enclosed (Supplemental material 2).

Stage three: Study selection

When searches have been applied in the databases, results will be imported to EndNote[51] giving an overview of the net selection and removing duplicates. The results will then be imported into Rayaan,[52] a suitable tool to facilitate the screening process of collaborative literature reviews. The first (OMN) and last author (GB) will review 5 – 10% of the gross selection, ensuring an agreed approach for deciding ineligible and eligible papers. To help guide the title and abstract screening phase, a screening tool will be developed[53] as eligible criteria should be detailed in early stages of developing a literature review,[36] in addition, the Rayyan interface will use PICoS filters to aid the screening process. During the title/abstract screening phase, we plan to have a "sifting" approach, narrowing down the net selection, resolving conflicts in Rayyan[52] in combination with frequent meetings as we narrow down

For peer review only

the number of papers. This approach has been successful in other literature reviews.[54] Upon completion of the study selection phase, the research team will evaluate the eligible papers' suitability before assessment and data charting and evaluate the data extraction chart. As Polanin and colleagues state[53] is the team approach important to promote an effective screening process. The process will be recorded, and the searches will be accounted for by PRISMA flowchart.[55]

Stage four: Data charting

Eligible papers subject to full-text assessment will be summarised in an extraction chart based on Arksey and O'Malley's template.[34] Additionally, the assessment of methodological quality using critical assessment tools can aid in identifying papers with poor methodological rigour. Identification of such papers may suggest that any results and findings are based on research with improper declaration of research methodology; as such, any generalisable results will be used cautiously. Although a quality assessment was not initially recommended by Arksey and O'Malley, later developments have seen quality assessment as a necessary component.[34, 37, 39] The critical appraisal assessment will be based on the critical appraisal skills programme (CASP)[56] and the mixed methods appraisal tool (MMAT).[57] An additional implementation of Weingarten, Paul and Leibovici's[58] ethical assessment for systematic reviews will aid us in illuminating ethical aspects within the net selection of papers. The first, second and last author will pilot 20% of the eligible papers to *'ensure that all necessary data is captured appropriately'* (p. 6),[36] and if needed, revise the data extraction chart using Microsoft Excel.[59] Studies with poor methodological quality and reporting of ethical and methodological standards will be discussed by the team. The planned items for extraction is presented in table 2.

Table 2. Tentative data for extraction chart

Items
ID number
Author
Year
Aim, objective and/or research questions
Does paper meet inclusion?
Does paper answer scoping aim question 1 or 2?
Research design
Context
Sample (who and how many)
Data collection

Data analysis
Summary of findings
If any, descriptions of interventions and outcomes
Quality appraisal
- For qualitative research: CASP [56]
- For mixed methods research and quantitative research: MMAT [57]

Description of ethics [58]
- Approved by a research ethical committee?
- Was informed consent obtained?
- Adequate data protection?
- Declaration of financial support?
- Declaration on potential conflicts of interest?

Stage five: collating, summarising, and reporting results

Arksey and O`Malley[34] highlight that the analytic framework used within scoping reviews should support an overview and presentation of a potentially large body of material (p. 28). A basic numerical analysis, mapping charts and tables in combination with a thematic analysis is common when collating, summarising, and reporting results within scoping reviews. Both Levac et al.[39] and Daudt et al.[37] states that applying meaning to the results as important, and especially understand the implications within a broader setting which can include both policy, contextual and practice implications. However, scoping reviews have been contested as failing to provide an actual scope of the research literature, and the high rejection rate among scoping reviews can be pinpointed to poor reporting, superficial presentation of results and lack of methodological guidance in this stage.[60-62] One effort to mitigate some of these issues was proposed by Bradbury-Jones et al.[35], further developing the reporting of results through adding clarity and focus on reporting patterns, advances, gaps, evidence for practice and research recommendations, presented as the PAGER-framework. This analytic approach can aid the research team by offering structure, identify inter-connections, identify advances and gaps and support reporting.[35] Besides utilizing the PAGER-framework, our analytical process will be aided by the recommendation of Levac et al.[39] suggesting a qualitative content analysis,[63] focusing on semantic and manifest descriptions to summarise the content as well as basic descriptive presentation of quantitative data[34] and visual displays.[36, 62] The research team will meet frequently at this stage, discussing results using key reflective questions as proposed by Bradbury-Jones et al.[35]

Stage six: consultation stage

For peer review only

This research project is situated within a larger research platform in Norway and Sweden. The research platform has its own patient, public and professional involvement (PPPI) group with yearly meetings. As such, results from stage five can be presented to this PPPI group.[39] Besides the PPPI group, the first author will present the results from stage five to a group of nursing researchers, students, and educators to discuss and further elaborate the findings. PPPI in health services research and within development of complex interventions have a central function to prioritise research questions, choosing useful research perspectives and evaluate results.[64, 65] The consultation stage in relation to scoping review methodology have been endorsed since its conception[34] as one way to gain additional information, transfer knowledge[66] and discuss questions for future research.[37] One example of PPPI in the developing stage of this scoping review is the collaboration with research librarians as proposed by Polloc et al.[67]

Patient and public involvement

Patients and/or the public were not involved in the design, conduct, reporting, or dissemination plans for this research, however, public, and professional involvement are planned in the consultation exercise of this scoping review.

ETHICS AND DISSEMINATION

Weingarten et al. advocated early for the need for ethical assessment in literature reviews,[58] while Wager and Wiffen state that researchers conducting literature reviews have an ethical responsibility, being transparent, accurate, identifying and reporting fraudulent research.[68] These research principles align with established ethical principles, namely reliability, honesty, respect, and accountability.[69] The development of a research protocol can make these guiding principles articulated in regard to respect to the methodology, reliability thorough the use of reporting guidelines and assessment tools and honesty and accountability through research integrity and collaboration with the before mentioned PPPI group. The planning of this scoping review is developed in unity with recommendations on ensuring value in research, with particular emphasis on appropriate research design, development of protocol and justifiable research priorities.[70]

For peer review only

This scoping review aims to map out literature concerning basic care, continuity of care and described models of care, patient care pathways and clinical practice guidelines targeting basic care among older people in long-term care. The results from this scoping review will support the initial phase of developing complex interventions in health services research. Besides identifying existing interventions, this scoping review will also take into consideration contextual circumstances, identify key uncertainties, and engage stakeholders.[65] Thus, the knowledge gained from this scoping review can serve as a theoretical underpinning for further development of an educational healthcare intervention aiming to promote basic care and continuity of care among older people in long-term care.

Through implementation of the reporting guidelines,[40, 41] assessment tools,[56-58] adherence to updated guidelines on the scoping review methodology,[35, 49] and a designated protocol,[71] the current review will be well equipped to make *'clear statements about where the specific focus should be and importantly, what does not require further research'* (p. 68),[72] thus reducing the probability of producing research waste.[73] Our research questions, search strategy, and comprehensive extraction chart can aid to prevent publication bias and selective reporting by having a broad approach to the literature field and eligible papers.

Acknowledgements

A great thanks goes to information specialists Annelie Ekberg-Andersson and Linda Borg at Karlstad University, Sweden, for providing guidance in how to develop a search strategy's in the course "systematic reviews in health sciences". Furthermore, a grateful appreciation to the members of the PPPI group who are engaged in the programmatic research platform "Continuity for Quality and Health" (CQCH) at Lovisenberg Diaconal University, Norway.

Contributors

All four authors were responsible for the study's inception and design. OMN wrote the initial draft. ERG, CO and HA participated with important intellectual content and critical revision. OMN and GB developed the search strategy, and GB supervised the study. All authors read and approved the final manuscript.

Funding

For peer review only

The authors have not declared a specific grant for this research from any funding agency in the public, commercial or not-for-profit sectors.

Competing interests

None of the authors declare any competing interests.

Patient consent for publication

Not required

References

1. Crawford R, Stoye G, Zaranko B. Long-term care spending and hospital use among the older population in England. *Journal of Health Economics* 2021;78:102477. doi: https://doi.org/10.1016/j.jhealeco.2021.102477
2. Walsh B, Wren M-A, Smith S, et al. An analysis of the effects on irish hospital care of the supply of care inside and outside the hospital. Dublin: Economic & Social Research Institute, 2019.
3. Gautun H, Syse A. Earlier hospital discharge: a challenge for Norwegian municipalities. *Nordic Journal of Social Research* 2017;8 doi: 10.7577/njsr.2204
4. National Institute of Aging. Aging in place: growing older at home 2017 [Available from: https://www.nia.nih.gov/health/aging-place-growing-older-home accessed 18.01 2022.
5. National Center for Health Statistics. Long-term care providers and services users in the United States, 2015-2016. In: National Center for Health S, ed. Hyattsville, MD, 2019.
6. Social Protection Committee and the European Commission. Long-term care report - Trends, challenges and opportunities in an agning society. Luxembourg, 2021.
7. National Institute on Aging. Enabling the future provision of long-term care in Canada. Toronto, 2019.
8. Karam M, Chouinard MC, Poitras ME, et al. Nursing Care Coordination for Patients with Complex Needs in Primary Healthcare: A Scoping Review. *Int J Integr Care* 2021;21(1):16. doi: 10.5334/ijic.5518
9. Ng R, Lane N, Tanuseputro P, et al. Increasing Complexity of New Nursing Home Residents in Ontario, Canada: A Serial Cross-Sectional Study. *J Am Geriatr Soc* 2020;68(6):1293-300. doi: 10.1111/jgs.16394
10. Næss G, Kirkevold M, Hammer W, et al. Nursing care needs and services utilised by home-dwelling elderly with complex health problems: observational study. *BMC Health Services Research* 2017;17(1):645. doi: 10.1186/s12913-017-2600-x
11. Zwakhalen SMG, Hamers JPH, Metzelthin SF, et al. Basic nursing care: The most provided, the least evidence based - A discussion paper. *J Clin Nurs* 2018;27(11-12):2496-505. doi: 10.1111/jocn.14296
12. Kitson A, Conroy T, Wengstrom Y, et al. Defining the fundamentals of care. *Int J Nurs Pract* 2010;16(4):423-34. doi: 10.1111/j.1440-172X.2010.01861.x

For peer review only

13. Henderson V. Basic principles of nursing care. Washington: American Nurses Publishing 1997.
14. Nightingale F. Notes on nursing - what it is, and what it is not. Philadelphia: Wolters Kluwer 2020.
15. Feo R, Conroy T, Jangland E, et al. Towards a standardised definition for fundamental care: A modified Delphi study. *J Clin Nurs* 2018;27(11-12):2285-99. doi: 10.1111/jocn.14247
16. Chatindiara I, Allen J, Hettige D, et al. High prevalence of malnutrition and frailty among older adults at admission to residential aged care. *Journal of Primary Health Care* 2020;12(4):305-17. doi: https://doi.org/10.1071/HC20042
17. Alves S, Teixeira L, Azevedo MJ, et al. Unmet needs in Primary Care of older clients with mental health concerns. *Int J Qual Health Care* 2019;31(4):312-18. doi: 10.1093/intqhc/mzy154
18. Schönfelder W, Eggebø H, Munkejord MC. Social care for older people – a blind spot in the Norwegian care system. *Social Work in Health Care* 2020;59(9-10):631-49. doi: 10.1080/00981389.2020.1847747
19. Groves A, Thomson D, McKellar D, et al. The Oakden Report. Adelaide: South Australia, Department for Health and Ageing, 2017.
20. Kalánková D, Kirwan M, Bartoníčková D, et al. Missed, rationed or unfinished nursing care: A scoping review of patient outcomes. *Journal of Nursing Management* 2020;28(8):1783-97. doi: https://doi.org/10.1111/jonm.12978
21. Scott PA, Harvey C, Felzmann H, et al. Resource allocation and rationing in nursing care: A discussion paper. *Nurs Ethics* 2019;26(5):1528-39. doi: 10.1177/0969733018759831
22. Lehto-Niskala V, Jolanki O, Jylhä M. Family's role in long-term care—A qualitative study of Finnish family members' experiences on supporting the functional ability of an older relative. *Health & Social Care in the Community* 2021;n/a(n/a) doi: https://doi.org/10.1111/hsc.13700
23. Kalánková D, Stolt M, Scott PA, et al. Unmet care needs of older people: A scoping review. *Nursing Ethics* 2021;28(2):149-78. doi: 10.1177/0969733020948112
24. Gustafsson N, Leino-Kilpi H, Prga I, et al. Missed Care from the Patient's Perspective - A Scoping Review. *Patient preference and adherence* 2020;14:383-400. doi: 10.2147/PPA.S238024
25. Strandell R. Care workers under pressure – A comparison of the work situation in Swedish home care 2005 and 2015. *Health & Social Care in the Community* 2020;28(1):137-47. doi: https://doi.org/10.1111/hsc.12848
26. Glette MK, Røise O, Kringeland T, et al. Nursing home leaders' and nurses' experiences of resources, staffing and competence levels and the relation to hospital readmissions – a case study. *BMC Health Services Research* 2018;18(1):955. doi: 10.1186/s12913-018-3769-3
27. Astrain BA. Growing demand, precariousness and austerity: The long way to universal and quality long-term care in Europe. Brussels: AGE Platform Europe, 2018.
28. Hamel C, Garritty C, Hersi M, et al. Models of provider care in long-term care: A rapid scoping review. *PLOS ONE* 2021;16(7):e0254527. doi: 10.1371/journal.pone.0254527
29. Schmüdderich K, Kiwitt J, Palm R, et al. Core elements and potential of nurse-led care models in residential long-term care: A scoping review. *Journal of Clinical Nursing* 2022;n/a(n/a) doi: https://doi.org/10.1111/jocn.16231
30. Davidson P, Halcomb E, Hickman L, et al. Beyond the rhetoric: what do we mean by a 'model of care'? *Aust J Adv Nurs* 2006;23(3):47-55.

For peer review only

31. Facchinetti G, D'Angelo D, Piredda M, et al. Continuity of care interventions for preventing hospital readmission of older people with chronic diseases: A meta-analysis. *International Journal of Nursing Studies* 2020;101:103396. doi: 10.1016/j.ijnurstu.2019.103396
32. Baker R, Freeman GK, Haggerty JL, et al. Primary medical care continuity and patient mortality: a systematic review. London, 2020:e600-e11.
33. Ma C, McDonald MV, Feldman PH, et al. Continuity of Nursing Care in Home Health: Impact on Rehospitalization Among Older Adults With Dementia. *Medical Care* 2021;59(10):913-20. doi: 10.1097/mlr.0000000000001599
34. Arksey H, O'Malley L. Scoping studies: towards a methodological framework. *International Journal of Social Research Methodology* 2005;8(1):19-32. doi: 10.1080/1364557032000119616
35. Bradbury-Jones C, Aveyard H, Herber OR, et al. Scoping reviews: the PAGER framework for improving the quality of reporting. *International Journal of Social Research Methodology* 2021:1-14. doi: 10.1080/13645579.2021.1899596
36. Peters M, Marnie C, Tricco AC, et al. Updated methodological guidance for the conduct of scoping reviews. *JBI Evidence Synthesis* 2020;18(10)
37. Daudt H, Mossel C, Scott S. Enhancing the Scoping Study Methodology: A Large, Interprofessional Team's Experience with Arksey and O'Malley's Framework. *BMC Medical Research Methodology* 2013;13 doi: 10.1186/1471-2288-13-48
38. Munn Z, Peters MDJ, Stern C, et al. Systematic review or scoping review? Guidance for authors when choosing between a systematic or scoping review approach. *BMC Medical Research Methodology* 2018;18(1):143. doi: 10.1186/s12874-018-0611-x
39. Levac D, Colquhoun H, O'Brien KK. Scoping studies: advancing the methodology. *Implementation science* 2010;5(1):1-9. doi: 10.1186/1748-5908-5-69
40. Moher D, Shamseer L, Clarke M, et al. Preferred reporting items for systematic review and meta-analysis protocols (PRISMA-P) 2015 statement. *Systematic Reviews* 2015;4(1):1. doi: 10.1186/2046-4053-4-1
41. Tricco AC, Lillie E, Zarin W, et al. PRISMA Extension for Scoping Reviews (PRISMA-ScR): Checklist and Explanation. *Annals of Internal Medicine* 2018;169(7):467-73. doi: 10.7326/M18-0850
42. Saunes IS, Karanikolos M, Sagan A. Norway - Health system review. Oslo: Norwegian Institute of Public Health 2020.
43. Wysocki A, Butler M, Kane RL, et al. AHRQ Comparative Effectiveness Reviews. Long-Term Care for Older Adults: A Review of Home and Community-Based Services Versus Institutional Care. Rockville (MD): Agency for Healthcare Research and Quality (US) 2012.
44. National Institute on Aging. What is long-term care? 2017 [Available from: https://www.nia.nih.gov/health/what-long-term-care accessed 13.01.22.
45. World Health Organization. Continuity and coordination of care: a practice brief to support implementation of the WHO Framework on integrated people-centred health services. Geneva: World Health Organization 2018:68 p.
46. OECD. Health at a glance 2021: OECD Indicators. 2021
47. Chu CH, McGilton KS, Spilsbury K, et al. Strengthening International Research in Long-Term Care: Recommended Common Data Elements to Support Clinical Staff Training. *Gerontology and Geriatric Medicine* 2021;7:2333721421999312. doi: 10.1177/2333721421999312
48. Methley AM, Campbell S, Chew-Graham C, et al. PICO, PICOS and SPIDER: a comparison study of specificity and sensitivity in three search tools for qualitative

For peer review only

systematic reviews. *BMC Health Services Research* 2014;14(1):579. doi: 10.1186/s12913-014-0579-0
49. Peters MDJ, Marnie C, Tricco AC, et al. Updated methodological guidance for the conduct of scoping reviews. *JBI Evidence Synthesis* 2020;18(10):2119-26. doi: 10.11124/jbies-20-00167
50. Bramer WM, de Jonge GB, Rethlefsen ML, et al. A systematic approach to searching: an efficient and complete method to develop literature searches. *Journal of the Medical Library Association : JMLA* 2018;106(4):531-41. doi: 10.5195/jmla.2018.283 [published Online First: 2018/10/01]
51. EndNote [program]. 20 version. Philadelphia, PA: Clarivate, 2022.
52. Ouzzani M, Hammady H, Fedorowicz Z, et al. Rayyan - a web and mobile app for systematic reviews. *Systematic Reviews* 2016;2016(5):210. doi: 10.1186/s13643-016-0384-4
53. Polanin JR, Pigott TD, Espelage DL, et al. Best practice guidelines for abstract screening large-evidence systematic reviews and meta-analyses. *Res Synth Methods* 2019;10(3):330-42. doi: 10.1002/jrsm.1354
54. Appelgren M, Bahtsevani C, Persson K, et al. Nurses' experiences of caring for patients with intellectual developmental disorders: a systematic review using a meta-ethnographic approach. *BMC Nursing* 2018;17(1):51. doi: 10.1186/s12912-018-0316-9
55. Page MJ, Moher D, Bossuyt PM, et al. PRISMA 2020 explanation and elaboration: updated guidance and exemplars for reporting systematic reviews. *BMJ* 2021;372:n160. doi: 10.1136/bmj.n160
56. CASP. Critical Apprasial Skills Programme qualitative checklist. Available at https://casp-uk.b-cdn.net/wp-content/uploads/2018/01/CASP-Qualitative-Checklist-2018.pdf. 2019
57. Hong QN, Fàbregues S, Bartlett G, et al. The Mixed Methods Appraisal Tool (MMAT) version 2018 for information professionals and researchers. *Education for Information* 2018;34:1-7. doi: 10.3233/EFI-180221
58. Weingarten MA, Paul M, Leibovici L. Assessing ethics of trials in systematic reviews. *BMJ* 2004;328(7446):1013-14. doi: 10.1136/bmj.328.7446.1013
59. Microsoft Excel for Mac [program]. 16.66.1 version, 2022.
60. Pham MT, Rajić A, Greig JD, et al. A scoping review of scoping reviews: advancing the approach and enhancing the consistency. *Res Synth Methods* 2014;5(4):371-85. doi: 10.1002/jrsm.1123
61. Tricco AC, Lillie E, Zarin W, et al. A scoping review on the conduct and reporting of scoping reviews. *BMC Medical Research Methodology* 2016;16(1):15. doi: 10.1186/s12874-016-0116-4
62. Khalil H, Peters MD, Tricco AC, et al. Conducting high quality scoping reviews-challenges and solutions. *J Clin Epidemiol* 2021;130:156-60. doi: 10.1016/j.jclinepi.2020.10.009
63. Braun V, Clarke V. Thematic analysis - a practical guide. London: Sage 2021.
64. Richards DA. The critical importance of patient and public involvement for research into complex interventions. In: Richards DA, Hallberg IR, eds. Complex interventions in health. New York: Routhledge 2015:46 - 50.
65. Skivington K, Matthews L, Simpson SA, et al. Framework for the development and evaluation of complex interventions: gap analysis, workshop and consultation-informed update. *Health Technol Assess* 2021;25(57):1-132. doi: 10.3310/hta25570

For peer review only

66. Dobrowolska B, Chruściel P, Pilewska-Kozak A, et al. Doctoral programmes in the nursing discipline: a scoping review. *BMC Nursing* 2021;20(1):228. doi: 10.1186/s12912-021-00753-6
67. Pollock D, Davies EL, Peters MD, et al. Undertaking a scoping review: A practical guide for nursing and midwifery students, clinicians, researchers, and academics. *Journal of advanced nursing* 2021;77(4):2102-13.
68. Wager E, Wiffen PJ. Ethical issues in preparing and publishing systematic reviews. *Journal of Evidence-Based Medicine* 2011;4(2):130-34. doi: https://doi.org/10.1111/j.1756-5391.2011.01122.x
69. All European Academies (ALLEA). The European Code of Conduct for Research Integrity. Berlin: Academy of Sciences and Humanities, 2017:20.
70. Ensuring Value in Research. Guiding principles Southampton: EVIR; 2022 [Available from: https://evir.org/our-principles/ accessed 10.06.2022.
71. Cullum N, Dumville J. Systematic reviews of the effects of interventions. In: Richards DA, Hallberg IR, eds. Complex interventions in health. Oxon: Routhledge 2015.
72. Bradbury-Jones C, Aveyard H. The incomplete scope of scoping reviews: A framework for improving the quality of reporting. *Journal of Clinical Nursing* 2021;30(21-22):e67-e68. doi: https://doi.org/10.1111/jocn.15998
73. Chalmers I, Glasziou P. Avoidable waste in the production and reporting of research evidence. *The Lancet* 2009;374(9683):86-89. doi: 10.1016/S0140-6736(09)60329-9

FIGURE CAPTION

Box1. Operationalisation of core concepts

Table 1. PICoS framework

Table 2. Tentative data for extraction chart

For peer review only

PRISMA-P 2015 Checklist

This checklist has been adapted for use with systematic review protocol submissions to BioMed Central journals from Table 3 in Moher D et al: Preferred reporting items for systematic review and meta-analysis protocols (PRISMA-P) 2015 statement. *Systematic Reviews* 2015 **4**:1

An Editorial from the Editors-in-Chief of *Systematic Reviews* details why this checklist was adapted - Moher D, Stewart L & Shekelle P: Implementing PRISMA-P: recommendations for prospective authors. *Systematic Reviews* 2016 **5**:15

Section/topic	#	Checklist item	Information reported		Line number(s)
			Yes	No	
**ADMINISTRATIVE INFORMATION**					
**Title**					
Identification	1a	Identify the report as a protocol of a systematic review	x		1-2
Update	1b	If the protocol is for an update of a previous systematic review, identify as such		x	N/A
**Registration**	2	If registered, provide the name of the registry (e.g., PROSPERO) and registration number in the Abstract	x		51
**Authors**					
Contact	3a	Provide name, institutional affiliation, and e-mail address of all protocol authors; provide physical mailing address of corresponding author	x		4 – 22<br><br>Only email for corresponding author is provided.
Contributions	3b	Describe contributions of protocol authors and identify the guarantor of the review	x		325 - 329
**Amendments**	4	If the protocol represents an amendment of a previously completed or published protocol, identify as such and list changes; otherwise, state plan for documenting important protocol amendments		x	N/A
**Support**					
Sources	5a	Indicate sources of financial or other support for the review	x		331 - 333
Sponsor	5b	Provide name for the review funder and/or sponsor		x	N/A
Role of sponsor/funder	5c	Describe roles of funder(s), sponsor(s), and/or institution(s), if any, in developing the protocol		x	N/A

BioMed Central The Open Access Publisher

Section/topic	#	Checklist item	Information reported Yes	Information reported No	Line number(s)
**INTRODUCTION**					
**Rationale**	6	Describe the rationale for the review in the context of what is already known	x		100 - 150
**Objectives**	7	Provide an explicit statement of the question(s) the review will address with reference to participants, interventions, comparators, and outcomes (PICO)	x		175 - 185
**METHODS**					
**Eligibility criteria**	8	Specify the study characteristics (e.g., PICO, study design, setting, time frame) and report characteristics (e.g., years considered, language, publication status) to be used as criteria for eligibility for the review	x		188 - 204
**Information sources**	9	Describe all intended information sources (e.g., electronic databases, contact with study authors, trial registers, or other grey literature sources) with planned dates of coverage	x		189 – 192
**Search strategy**	10	Present draft of search strategy to be used for at least one electronic database, including planned limits, such that it could be repeated	x		Supplemental material 2 + 199- 202
STUDY RECORDS					
Data management	11a	Describe the mechanism(s) that will be used to manage records and data throughout the review	x		207 – 209
Selection process	11b	State the process that will be used for selecting studies (e.g., two independent reviewers) through each phase of the review (i.e., screening, eligibility, and inclusion in meta-analysis)	x		207 - 222
Data collection process	11c	Describe planned method of extracting data from reports (e.g., piloting forms, done independently, in duplicate), any processes for obtaining and confirming data from investigators	x		225 – 240
**Data items**	12	List and define all variables for which data will be sought (e.g., PICO items, funding sources), any pre-planned data assumptions and simplifications	x		Table 2
**Outcomes and prioritization**	13	List and define all outcomes for which data will be sought, including prioritization of main and additional outcomes, with rationale		x	N/A
**Risk of bias in individual studies**	14	Describe anticipated methods for assessing risk of bias of individual studies, including whether this will be done at the outcome or study level, or both; state how this information will be used in data synthesis		x	N/A
DATA					
**Synthesis**	15a	Describe criteria under which study data will be quantitatively synthesized	x		245 - 265

BioMed Central
The Open Access Publisher

For peer review only - http://bmjopen.bmj.com/site/about/guidelines.xhtml

Section/topic	#	Checklist item	Information reported Yes	Information reported No	Line number(s)
	15b	If data are appropriate for quantitative synthesis, describe planned summary measures, methods of handling data, and methods of combining data from studies, including any planned exploration of consistency (e.g., I^2, Kendall's tau)		x	N/A
	15c	Describe any proposed additional analyses (e.g., sensitivity or subgroup analyses, meta-regression)		x	N/A
	15d	If quantitative synthesis is not appropriate, describe the type of summary planned	x		260 - 263
**Meta-bias(es)**	16	Specify any planned assessment of meta-bias(es) (e.g., publication bias across studies, selective reporting within studies)	x		310 - 316
**Confidence in cumulative evidence**	17	Describe how the strength of the body of evidence will be assessed (e.g., GRADE)	x		232 - 234

For peer review only

BioMed Central The Open Access Publisher

Additional file 2

Search blocks PubMed – tested 28.10.2022

Nr	Population - Older people	Mesh & All Fields
#1	Aged [MT]	3.420.178
#2	Elderly [MT]	3.420.178
#3	Older adult* [AF]	279.685
#4	Old [AF]	1.231.066
#5	Older people [AF]	421.434
#6	Senior* [AF]	89.018
#7	((((((#1) OR (#2)) OR (#3)) OR (#4)) OR (#5)) OR (#6)	4.659.920

Nr	Population - Relatives	Mesh & All Fields
#8	Family [MT]	358.654
#9	Spouse* [MT]	18.609
#10	Daughter [AF]	238.910
#11	Son [AF]	254.242
#12	Relatives [AF]	2.964.050
#13	Significant other* [AF]	900.106
#14	Kinship [AF]	5.269
#15	Next of kin [AF]	2.109
#16	Informal caregiver* [AF]	27.832
#17	((((((((((#8) OR (#9)) OR (#10)) OR (#11)) OR (#12)) OR (#13) OR (#14)) OR (#15) OR (#16))	3.801.433

Nr	Population - Nursing staff	Mesh & All Fields
#18	Allied health professional* [MT]	5.326
#19	Licenced practical nurse [MT]	920
#20	Nurse*[MT]	160.052
#21	Registered nurse* [AF]	20.593
#22	Licensed practice nurse* [AF]	3.031
#23	Registered practical nurse* [AF]	9.898
#24	Licensed vocational nurse* [AF]	335
#25	Nurse assistant* [AF]	16.486
#26	Health care assistant [AF]	71.365
#27	Formal caregiver [AF]	3.333
#28	Health care professional [AF]	798.411
#29	Practicing nurse* [AF]	123.426
#30	Professional nurs* [AF]	121.812
#31	Associate professional nurs* [AF]	21.911
#32	Nursing aide* [AF]	3.152
#33	Nursing assistant* [AF]	18.987
#34	Nurse specialist* [AF]	16.885
#35	((((((((((((#18) OR (#19) OR (#20) OR (#21)) OR (#22)) OR (#23)) OR (#24)) OR (#25) OR (#26)) OR (#27) OR (#28) OR (#29)) OR (#30)) OR (#31) OR (#32)) OR (#33) OR (#34)	961.356

Nr	Phenomenon of Interest - Continuity of care	Mesh & All Fields
#36	Continuity of patient care [MT]	281.675
#37	Continuity of care [AF]	293.204
#38	Continuity of nursing care [AF]	69.610
#39	Care continuity [AF]	293.204
#40	Informational continuity [AF]	131.068
#41	Relational continuity [AF]	250.791
#42	Interpersonal continuity [AF]	5.732
#43	Longitudinal continuity [AF]	27.290
#44	Management continuity [AF]	244.955
#45	((((((((((#36)) OR (#37)) OR (#38)) OR (#39)) OR (#40)) OR (#41) OR (#42)) OR (#43) OR (#44))	785.571

For peer review only

Additional file 2

Nr	Phenomenon of Interest - Care & nursing	Mesh & All Fields
#46	Nursing care [MT]	140.537
#47	Nursing [MT]	261.792
#48	Patient* care need* [AF]	354.288
#49	Basic care [AF]	58.270
#50	Essence of care [AF]	2.467
#51	Essential care [AF]	86.533
#52	Fundamental care [AF]	22.069
#53	Eldercare [AF]	578
#54	Elderly care [AF]	77.036
#55	Geriatric care [AF]	69.432
#56	Care of older persons [AF]	118.422
#57	Care of elderly persons [AF]	861.412
#58	Care of aged persons [AF]	878.385
#59	Old age assistance [AF]	2.209
#60	Care [AF]	3.062.962
#61	Care need* [AF]	575.293
#62	Physical care need* [AF]	64.176
#63	Basic nursing need* [AF]	16.847
#64	Physiological need* [AF]	73.983
#65	Biological need* [AF]	415.636
#66	Psychosocial need* [AF]	34.275
#67	Physical care [AF]	283.388
#68	Advanced care [AF]	176.140
#69	Support [AF]	11.231.769
#70	Relationship* [AF]	2.441.775
#71	Caring network [AF]	3.869
#72	Care plan [AF]	63.001
#73	Compassionate care [AF]	3.875
#74	Core nursing [AF]	9.802
#75	(((((((((((#46) OR (#47)) OR (#48) OR (#49) OR (#50) OR (#51) OR (#52)) OR (#53)) OR (#54)) OR (#55)) OR (#56) OR (#57)) OR (#58) OR (#59)) OR (#60) OR (#61) OR (#62)) OR (#63) OR (#64)) OR (#65) OR (#66) OR (#67) OR (#68)) OR (#69) OR (#70) OR (#71) OR (#72) OR (#73) OR (#74)	14.763.344

Nr	Phenomenon of Interest - Models, frameworks and interventions of care	Mesh & All Fields
#76	Nursing model [MT]	12.223
#77	Nursing process [MT]	130.423
#78	Critical pathway [MT]	7.617
#79	Clinical protocol [MT]	186.684
#80	Practice guideline [MT]	137.148
#81	Care model* [AF]	436.255
#82	Nursing framework* [AF]	23.558
#83	Care framework* [AF]	71.183
#84	Nursing approach [AF]	70.370
#85	Care approach [AF]	318.776
#86	Nursing programme [AF]	114.350
#87	Care programme [AF]	427.512
#88	Nursing intervention* [AF]	101.600
#89	Nurse-led [AF]	4.778
#90	Patient care pathway [AF]	27.079
#91	Patient pathway [AF]	250.485
#92	Care pathway [AF]	99.710
#93	Clinical pathway [AF]	200.413
#94	Clinical recommendation* [AF]	142.054
#95	Clinical path [AF]	84.521
#96	Clinical care plan [AF]	24.467
#97	Care path [AF]	8.899
#98	Care guideline [AF]	106.599
#99	Care recommendation* [AF]	110.130
#100	Care protocol [AF]	1.156
#101	(((((((((((((((((((((((((((((#76)) OR (#77) OR (#78)) OR (#79) OR (#80) OR (#81)) OR (#82) OR (#83)) OR (#84) OR (#85) OR (#86) OR (#87) OR (#88)) OR (#89) OR (#90) OR (#91) OR (#92) OR (#93) OR (#94)) OR (#95) OR (#96) OR (#97) OR (#98) OR (#99) OR (#100)	2.023.126

For peer review only

Additional file 2

Nr	Context - Long term care, home healthcare, home nursing, and other care facilities	Mesh & All Fields
#102	Community health services [MT]	326.981
#103	Home health services [MT]	124.187
#104	Home health nursing [MT]	375
#105	Long term care [MT]	27.995
#106	Primary health care [MT]	186.101
#107	Nursing home* [MT]	43.534
#108	Housing for the elderly [MT]	1.652
#109	Housing [MT]	36.116
#110	Primary care [AF]	554.734
#111	Community care [AF]	322.329
#112	Community health services [AF]	432.422
#113	Municipal care [AF]	59.539
#114	Home care [AF]	941.943
#115	Home dwelling [AF]	29.464
#116	Home healthcare [AF]	72.406
#117	Home and community-based care [AF]	5.116
#118	Home and community-based services [AF]	4.397
#119	Longitudinal care [AF]	60.893
#120	Assisted living [AF]	29.473
#121	Independent living [AF]	36.609
#122	Residential care aged facilities [AF]	4.229
#123	Sheltered accommodation [AF]	300
#124	Sheltered housing [AF]	2.541
#125	Residential care [AF]	15.975
#126	Care home [AF]	158.055
#127	Day care [AF]	169.885
#128	Special accommodation [AF]	17.246
#129	((((((((((((((((((((((((((((#102)) OR (#103)) OR (#104) OR (105)) OR (#106) OR (#107)) OR (#108) OR (#109) OR (#110)) OR (#111) OR (#112)) OR (#113) OR (#114) OR (#115) OR (#116) OR (#117)) OR (#118) OR (#119) OR (#120) OR (#121) OR (#122) OR (#123)) OR (#124) OR (#125) OR (#126)) OR (#127) OR (#128)	2.067.109

For peer review only

BMJ Open

Fundamental Nursing Care Focusing Older Peoples Needs and Continuity of Long-Term Care: A Scoping Review Protocol

Journal:	*BMJ Open*
Manuscript ID	bmjopen-2022-069798.R1
Article Type:	Protocol
Date Submitted by the Author:	13-Mar-2023
Complete List of Authors:	Nordaunet, Ole Martin; Karlstad University; Lovisenberg Diaconal University College<br>Gjevjon, Edith; Lovisenberg Diaconal University College, Bachelor Education in Nursing<br>Olsson, Cecilia; Karlstad University<br>Aagaard , Hanne; Lovisenberg Diaconal University College<br>Borglin, Gunilla; Lovisenberg Diaconal University College, Department of Nursing
**Primary Subject Heading**:	Nursing
Secondary Subject Heading:	Health services research
Keywords:	Quality in health care < HEALTH SERVICES ADMINISTRATION & MANAGEMENT, PRIMARY CARE, STATISTICS & RESEARCH METHODS, Aged

SCHOLARONE™
Manuscripts

Fundamental Nursing Care Focusing Older Peoples Needs and Continuity of Long-term Care: A Scoping Review Protocol

Nordaunet O.M., RN, MSc, PhD-student [1, 2] Gjevjon E. R., RN, MSc, PhD, Professor of Nursing[1] Olsson C., RN, MSc, PhD, Associate Professor[2] Aagaard H., RN, MSc, PhD, Professor[1,2] Borglin G., RN, MSc, PhD, Professor of Nursing[1]

[1]Lovisenberg Diaconal University College, Department of Bachelor Education (Nursing). Lovisenberggata 15B, NO-0456 Oslo, Norway

[2] Karlstad University, Institute of Health Sciences, Department of Nursing. Universitetsgatan 2, 651 88 Karlstad, Sweden

Ole Martin Nordaunet:	https://orcid.org/0000-0002-6061-0033
Edith Roth Gjevjon:	https://orcid.org/0000-0002-9656-522X
Cecilia Olsson:	https://orcid.org/0000-0002-0944-5650
Hanne Aagaard:	https://orcid.org/0000-0002-7810-9298
Gunilla Borglin:	https://orcid.org/0000-0002-7934-6949

Word count: 2320 excl. 2 Tables and 1 box

Corresponding author:

Ole Martin Nordaunet

For peer review only - http://bmjopen.bmj.com/site/about/guidelines.xhtml

Lovisenberg Diaconal University College,

Lovisenberggata 15b, 0456 Oslo

Norway

E-mail: Ole.martin.nordaunet@ldh.no

Phone: + 47 997 313 70

For peer review only

ABSTRACT

Introduction: Knowledge about long-term care services ability, regardless of if the service is home-based or facility based, to provide an optimal and comprehensive fundamental nursing care (understood as focusing on physical, relational, and psychosocial needs) consistently over time is sparse. Research into nursing indicate the presence of a discontinuous and fragmented health care service, and that fundamental nursing care such as mobilisation, nutrition, and hygiene among older people (65 years and above) seems to be, regardless of reasons, systematically rationed by nursing staff. Thus, our scoping review aims to explore the published scientific literature on fundamental nursing care and continuity of care targeting older people's needs while also describing identified nursing interventions with the same foci in a long-term care context.

Method and analysis: The upcoming scoping review will be conducted in accordance with Arksey and O'Malley's methodological framework for scoping studies. Search strategies will be developed and adjusted to each database e.g., PubMed, CINAHL and PsychINFO. Searches will be limited to the years of 2002 – 2023. Studies focusing our aim will regardless of study design be eligible for inclusion. Included studies will be quality assessed and data will be charted using an extraction form. Textual data will be presented through a thematic analysis and numerical data by a descriptive numerical analysis. This protocol adheres to the PRISMA protocol checklist.

Ethics and dissemination: The upcoming scoping review will take into consideration ethical reporting in primary research as part of the quality assessment. The findings will be submitted to an open-access peer-reviewed journal. Under the Norwegian act on medical and health-related research this study does not need ethical clearance by a Regional Ethical Review Authority as it will not generate any primary data or obtain sensitive data or biological samples.

Registration: Open Science Framework: https://doi.org/10.17605/OSF.IO/XJ39E

For peer review only

STRENGTHS AND LIMITATION OF THE STUDY

- The combination of quality assessment, reporting guidelines and a well-established methodology will support 'clear statements about where the

For peer review only - http://bmjopen.bmj.com/site/about/guidelines.xhtml

specific focus should be and importantly, what does not require further research.

- Including qualitative, quantitative, and mixed methods design will assist us to achieve a comprehensive picture of the present knowledgebase.
- Excluding grey literature might limit our results.

For peer review only

INTRODUCTION

As the older part of the population is increasing and living longer, more and more older people find themselves in need of long-term care.[1] Research implies that the demand for long-term care will rise substantially all over Europe.[2] Nowadays older people are also more often described to have complex care needs mainly due to high age and multiple chronic conditions. Consequently, they are correspondingly described as requiring comprehensive fundamental nursing care, related to nutrition, mobility, elimination, personal hygiene etc.[3-5] Attending to such needs lies well within the remit of nursing. Particularly as we like Henderson[6] recognise fundamental nursing care to be directed at assisting with a person´s personal needs such as nutrition, cleanliness, elimination, mobility etc. Thus, "assisting people to do things they would normally do for themselves if only they were able" (p.149).[7] Fundamental nursing care are described as to combining physical, relational, and psychosocial dimensions of nursing care encounters.[8, 9] Research[10, 11] has described fundamental nursing care to be in a poor condition resulting in findings unfit to provide any evidence-based guidance. In-depth knowledge about the scope and practice of fundamental nursing care and of the practice setting are therefore of vital importance to support nursing staff's skills and abilities in delivering an optimal fundamental nursing care to older people in need of help and support.

Most of the workforce responsible to deliver fundamental nursing care in the long-term care context, regardless of the service is homebased or facility based, consists

For peer review only - http://bmjopen.bmj.com/site/about/guidelines.xhtml

of nursing staff (registered, licensed, diploma nurses and nurse assistants). Some literature reviews highlights that the nursing staff within this increasingly highly specialised context need to represent a wide variety of competencies.[12, 13] Knowledge about long-term care services ability to provide an optimal and comprehensive care targeting fundamental nursing care (understood as physical, relational, and psychosocial needs) consistently (i.e., continuity of care) over time is sparse.[14-15] Continuous care (i.e., continuity of care) is often described as a key-indicator for the concept of quality of care. As a key-indicator, it is known to prevent unnecessary hospital admissions, lowering cost as well as decreasing mortality.[16-18] Ensuring continuity of care impacts the quality of care and vice versa. Moreover, research into nursing suggest the presence of a discontinuous and fragmented health care service, and that needs such as mobilisation, nutrition and hygiene among older people are likely to become rationed by nursing staff.[19-20] Thus, unmet, or rationed care is not uncommon in the long-term care context.[20-21]

It has been described that nursing staff working in accordance with distinctly articulated nursing interventions such as different well-defined models of care, patient care pathways or in accordance with clinical practice guidelines are more likely to work systematically and towards mutually set goals in care[3]. Hence the deliverance of care can more easily be assessed and evaluated on a regular basis. Implementing such strategies to care which includes structured assessments and evaluations might be one way to aid nursing staff to deliver fundamental nursing care in a continuous

For peer review only - http://bmjopen.bmj.com/site/about/guidelines.xhtml

and optimal way. It could also promote fundamental nursing care in a more predictable manner that corresponds to older patients' preferences and needs continuously over time. However, recent literature reviews suggest that the implementation of distinctly articulated and well-defined nursing interventions (understood as above description) targeting fundamental nursing care and/or continuity of care among older people in long-term care is scant.[22-23] Consequently, the development and testing of viable nursing interventions supporting nursing staff skills and abilities in relation to the deliverance of a continuously and optimal fundamental nursing care among older people are vital in the long-term care context to ensure both continuity- and quality of care. Accordingly, our scoping review aims to explore the published scientific literature on fundamental nursing care and continuity of care targeting older people's needs while also describing identified nursing interventions with the same foci (box 1) in a long-term care context.

METHODS

Our scoping review will be based on Arksey and O'Malley's[24] methodological framework as well as on updated methodology.[25-27] Scoping review methodology is particularly useful in assessing key characteristics and identifying knowledge gaps in the literature within complex topics.[24, 28, 29] Two authors (ERG & GB) will collaborate closely with the first author (OMN) during the process, while two authors (HA & CO) will provide critical assessment and feedback within the overall process of stages. This protocol is designed and reported in accordance with the preferred

For peer review only - http://bmjopen.bmj.com/site/about/guidelines.xhtml

reporting items for the systematic review and meta-analysis protocols checklist, PRISMA-P (Supplemental material 1).[30] Our upcoming scoping review will adhere to the PRISMA-Scoping Review checklist[31] and is registered at Open Science Framework, last updated the 9th of March 2023 (https://doi.org/10.17605/OSF.IO/XJ39E).

Box 1. Operationalisation of tentative core concepts in the review

Long-term care are here operationalised as:

- Health care delivered over prolonged periods of time in the patient's home, nursing home or sheltered housing, but can as well be delivered in other contexts in the community, such as, but not limited to day care centres, assisted living facilities and retirement homes[1, 32-34]

Fundamental nursing care are here operationalised as:

- Care directed at assisting with a person's personal needs such as mobility, nutrition, cleanliness etc. combined with physical, psychosocial, and relational dimensions[8, 9]

Continuity of care are here operationalised as:

- 'The degree to which a series of discrete health care events is experienced by people as coherent and interconnected over time and consistent with their health needs and preferences' (p. 8)[35]

Key stakeholders are here operationalised as:

- Older people and nurses

Nurses and/or nursing staff are here operationalised as:

- Practicing nurses, professional nurses, associate professional nurses, healthcare assistants, nursing aides, nurse specialists, registered nurses, registered practical, licenced practice nurses, hereafter referred to as nurses[36, 37]

Nursing interventions are here operationalised as:

- As distinctly articulated and defined nursing activities, models of care, patient care pathways or clinical practice guidelines (c.f.)[38]

Stage one: Identifying the research questions

For peer review only - http://bmjopen.bmj.com/site/about/guidelines.xhtml

The tentative questions to the literature for our upcoming scoping review are deliberately broad to cover the breadth of research evidence in our field of foci. Thus, we are aiming for comprehensiveness. One of the strengths with scoping reviews is the iterative methodological process which will allow us to decide to add supplementary questions or to fine tune our suggested questions based on the findings emerging during the review process.[24] A modified PICOS (Population, Intervention, Comparision, Outcome and Study Setting) will support us in our searchers (Table 1) and in deciding the suitability of our tentative questions to the literature. [39, 40]

Table 1. Framework for determine eligibility of the tentative research questions

Criteria	Determinants
**P**opulation	Older people (65 years and above), nurses (box 1) and relatives in a broad understanding
**I**ntervention (phenomenon of interest)	Fundamental nursing care and nursing interventions (box 1) targeting older people's needs
**C**omparison	*NA*
**O**utcome	Physical, relational, or psychosocial needs<br>Continuity of care<br>Nursing Interventions targeting continuity and/or fundamental nursing care
**S**tudy setting	Home-based or facility based long-term care (box 1) for older people

For peer review only

Our upcoming scoping review will tentatively explore the following questions to the literature:

- What fundamental nursing care (box 1) are described in the literature as targeting older peoples need in a long-term care context?
- How are fundamental nursing care described and experienced by key-stake holders (box 1) in a long-term care context?
- What fundamental nursing care interventions (box 1) are described in the literature targeting older people's needs or their continuity of care in a long-term care context?

Additionally, sub-questions will be used to facilitate an in-depth exploration of the included papers and their findings. Sub-questions will tentatively encompass the following: what characterise the long-term care settings described, who's perspective is investigated (e.g., stakeholders), type of nurses delivering fundamental nursing care, which outcomes are in focus and country of origin.

Stage two: Identifying relevant studies

Searches will be conducted in the three databases PubMed, CINAHL and PsycINFO, covering the majority of published peer- reviewed health service research. The search strategies will be developed following PICOS (Table 1). The first author consulted an information specialist at Karlstad University, Sweden to refine the initial tentative

For peer review only

search strategy (supplemental file 2). As part of the iterative process our search strategies will be further tested and refined before executing the upcoming scoping review. The final search strategies for each individual database will also be reviewed by an information specialist at Karlstad University, Sweden. To reflect the iterative approach of scoping reviews it is important to strike a balance between comprehensiveness and manageability of search results.[26] Accordingly, we will include controlled subject headings (MeSH), keywords, synonyms, and Boolean operators.[41] The searches will be time limited to 2002 – 2023. We chose to begin our searches year 2002 as initial test searchers indicated a lack of published peer-reviewed papers in relation to fundamental nursing care before this year. Included papers reference lists will be searched. [41-42] Considering what types of research likely to answering our tentative questions to the literature, but also resources and time needed to scan for grey-literature[42] only published peer-reviewed research in English will be included.

Stage three: Study selection

When searches have been applied in the databases, eligible papers will be imported to EndNote[43] giving us a clear overview of the net selection while also removing any duplicates. The remaining papers will then be imported into Rayaan.[44] The first (OMN) and last author (GB) will review 5 – 10% of the gross selection, ensuring an agreed approach for deciding ineligible and eligible papers. To help guide the title and abstract screening, the PICOS framework (Table 1) will be utilised to support the

For peer review only

process (c.f.)[45] mainly as the eligible criteria relates to our determinants in the framework. For example, all study designs are eligible, and our population will be limited to nurses (Box 1) and older people defined as 65 years and older. Our rationale for the latter is that 65 years is one of the standard cut-offs for older people in most research and databases. However, considering the iterative process of a scoping review, the eligibility criteria are likely to be further developed as our knowledge in the field accumulate.[24] The Rayyan interface will additionally use the PICOS framework (Table 1) to further support us in the screening process. Research type, language and ethical considerations will also be part of our eligible criteria. During the title and abstract screening, we plan to have a “sifting” approach, narrowing down the net selection, while resolving conflicts in Rayyan.[44] Frequent meetings are planned to support us in the sifting approach. This strategy has been described as a successful strategy by others.[46] Upon completion of the study selection, the research team will evaluate the eligible papers’ suitability before the assessment and data charting will begin. The process as whole will be recorded with a logbook and the search process will be illustrated using the PRISMA flowchart.[47]

For peer review only

Stage four: Data charting

Eligible papers subject to full-text assessment will be summarised in an extraction chart and the tentatively planned extraction data can be viewed in Table 2. Additionally, the assessment of methodological quality using critical assessment tools will aid us in identifying papers with poor methodological rigour. Identification of such papers may suggest that any results and findings are based on research with improper

For peer review only - http://bmjopen.bmj.com/site/about/guidelines.xhtml

declaration of research methodology; as such, any generalisable results will be used cautiously. Although a quality assessment was not initially recommended by the originators later developments have seen quality assessment as a necessary component.[24, 26, 28] Critical appraisal, that is quality assessment of included papers will be done in accordance with their study design. We will implement Weingarten et al[48] ethical assessment for systematic reviews. The first, second and last author will pilot 20% of the eligible papers to 'ensure that all necessary data is captured appropriately' (p. 6)[27], the data extraction chart will be revised after the piloting if needed. Eligible papers with questionable method or ethical reporting will be discussed by the team.

Table 2. Tentative data for extraction chart

Data
ID number
Author
Year
Country of origin
Aim, objective and/or research questions
Study Context and description/definition of long-term care
Key stakeholder (who)
How are "nurse" described/defined
Research design
Sample (who and how many)
Data collection
Data analysis
Summary of findings
Descriptions of components in nursing interventions and outcomes
Quality appraisal adjusted to research design
Description of ethics (48)<br>- Approved by a research ethical committee?<br>- Was informed consent obtained?

For peer review only

- Adequate data protection?
- Declaration of financial support?
- Declaration on potential conflicts of interest?

Stage five: collating, summarising, and reporting results

Arksey and O`Malley[24] highlight that the analytic framework used within scoping reviews should support an overview and presentation of a potentially large body of material (p. 28). A basic numerical analysis, mapping charts and tables in combination with a thematic analysis is common when collating, summarising, and reporting results within scoping reviews. Both Levac et al.[28] and Daudt et al.[26] states that applying meaning to the results as important, and especially understand the implications within a broader setting which can include both policy, contextual and practice implications. However, scoping reviews have been contested as failing to provide an actual scope of the research literature, and the high rejection rate among scoping reviews can be pinpointed to poor reporting, superficial presentation of results and lack of methodological guidance in this stage.[49-51] One effort to mitigate some of these issues was proposed by Bradbury-Jones et al.,[25] further developing the reporting of results through adding clarity and focus on reporting Patterns, Advances, Gaps, Evidence for practice and Research recommendations, presented as the PAGER-framework. This analytic approach can aid the research team by offering structure,

For peer review only - http://bmjopen.bmj.com/site/about/guidelines.xhtml

identify inter-connections, identify advances and gaps and support reporting.[25] Besides utilizing the PAGER-framework, our analytical process will be aided by the recommendation of Levac et al.[28] suggesting a qualitative thematic analysis[52], focusing on semantic and manifest descriptions to summarise the content as well as basic descriptive presentation of quantitative data[24] and visual displays .[27, 49] The research team will hold regular meetings discussing results using key reflective questions as proposed by Bradbury-Jones et al.[25]

Patient and public involvement

Patients and/or the public were not involved in the design, conduct, reporting, or dissemination plans for this research.

ETHICS AND DISSEMINATION

Under the Norwegian act on medical and health-related research[53] the upcoming scoping review does not need ethical clearance by a Regional Ethical Review Authority as it will not generate any primary data or obtain sensitive data or biological samples. It is still important to note that for example Weingarten et al.[48] advocated early on for the need for ethical assessment in literature reviews. This was later concurred by Wager and Wiffen[54] who states that researchers conducting literature reviews have an ethical responsibility, being transparent, accurate, identifying and reporting fraudulent research. These research principles align with established ethical principles, namely reliability, honesty, respect, and accountability.[55] The planning of this scoping review is developed in unity with recommendations on ensuring value in

For peer review only

research, with particular emphasis on appropriate research design, development of protocol and justifiable research priorities.[56]

The results from the upcoming scoping review will support us in the initial phase of the Medical Research Council framework for complex interventions.[57] Thus, the knowledge gained from the upcoming scoping review can serve as a theoretical underpinning for the possible development of an educational intervention targeting fundamental nursing care aiming at continuity of care and older people's needs in a long-term care setting.

In the upcoming scoping review, we will implement reporting guidelines,[30, 31] conduct quality assessments of included papers method and ethical reporting, adhere to updated guidelines on the scoping review methodology[25, 27] and use a designated protocol.[58] Thus, it is likely that the upcoming scoping review will support us in making 'clear statements about where the specific focus should be and importantly, what does not require further research'(p. 68).[59] Implementing such strategies can also help to reduce the probability of producing research waste.[60]

Acknowledgements

Our sincere gratitude goes to information specialists AEA and LB at Karlstad University, Sweden for their support in the development of our initial search strategies.

Contributors

For peer review only

All five authors were responsible for the study's inception and design. OMN wrote the initial draft. ERG, CO and HA contributed with important intellectual content and critical revision. OMN and GB developed the search strategy, and GB supervised the study. All authors read and approved the final manuscript.

Funding

The authors have not declared a specific grant for this research from any funding agency in the public, commercial or not-for-profit sectors.

Competing interests

None of the authors declare any competing interests.

Patient and public involvement

Not appliable

Patient consent for publication

Not required

References

1. Saunes IS, Karanikolos M, Sagan A. Norway - Health system review. Oslo: Norwegian Institute of Public Health; 2020.
2. Carrino L, Orso CE, Pasini G. Demand of long-term care and benefit eligibility across European countries. Health Economics. 2018;27(8):1175-88. doi: https://doi.org/10.1002/hec.3665
3. Karam M, Chouinard MC, Poitras ME, Couturier Y, Vedel I, Grgurevic N, et al. Nursing Care Coordination for Patients with Complex Needs in Primary Healthcare: A Scoping Review. Int J Integr Care. 2021;21(1):16. doi: 10.5334/ijic.5518

For peer review only

4. Næss G, Kirkevold M, Hammer W, Straand J, Wyller TB. Nursing care needs and services utilised by home-dwelling elderly with complex health problems: observational study. BMC Health Services Research. 2017;17(1):645. doi: 10.1186/s12913-017-2600-x
5. Ng R, Lane N, Tanuseputro P, Mojaverian N, Talarico R, Wodchis WP, et al. Increasing Complexity of New Nursing Home Residents in Ontario, Canada: A Serial Cross-Sectional Study. J Am Geriatr Soc. 2020;68(6):1293-300. doi: 10.1111/jgs.16394
6. Henderson V. The nature of nursing; a definition and its implications for practice, research, and education. New York: Macmillan; 1966.
7. Richards DA, Borglin G. 'Shitty nursing' – The new normal? International Journal of Nursing Studies. 2019;91:148-52. doi: https://doi.org/10.1016/j.ijnurstu.2018.12.018
8. Kitson A, Conroy T, Wengstrom Y, Profetto-McGrath J, Robertson-Malt S. Defining the fundamentals of care. Int J Nurs Pract. 2010;16(4):423-34. doi: 10.1111/j.1440-172X.2010.01861.x
9. Kitson A, Conroy T, Kuluski K, Locock L, Lyons R. Reclaiming and redefining the Fudamentals of Care: Nursing´s respons to meeting patients´ basic human needs. Adelaide: University of Adelaide; 2013.
10. Zwakhalen SMG, Hamers JPH, Metzelthin SF, Ettema R, Heinen M, de Man-Van Ginkel JM, et al. Basic nursing care: The most provided, the least evidence based - A discussion paper. J Clin Nurs. 2018;27(11-12):2496-505. doi: 10.1111/jocn.14296
11. Richards DA, Hilli A, Pentecost C, Goodwin VA, Frost J. Fundamental nursing care: A systematic review of the evidence on the effect of nursing care interventions for nutrition, elimination, mobility and hygiene. J Clin Nurs. 2018;27(11-12):2179-88. doi: 10.1111/jocn.14150
12. Rostad HM, Skinner MS, Hellesø R, Sogstad MKR. Towards specialised and differentiated long-term care services: a cross-sectional study. BMC Health Services Research. 2020;20(1):793. doi: 10.1186/s12913-020-05647-y
13. Rusli KDB, Tan AJQ, Ong SF, Speed S, Lau Y, Liaw SY. Home-based nursing care competencies: A scoping review. Journal of Clinical Nursing. 2021:1 - 15. doi: https://doi.org/10.1111/jocn.16169
14. Kitson AL. The Fundamentals of Care Framework as a Point-of-Care Nursing Theory. Nurs Res. 2018;67(2):99-107. doi: 10.1097/nnr.0000000000000271
15. Pentecost C, Frost J, Sugg HVR, Hilli A, Goodwin VA, Richards DA. Patients' and nurses' experiences of fundamental nursing care: A systematic review and qualitative synthesis. J Clin Nurs. 2020;29(11-12):1858-82. doi: 10.1111/jocn.15082
16. Baker R, Freeman GK, Haggerty JL, Bankart MJ, Nockels KH. Primary medical care continuity and patient mortality: a systematic review. London2020. p. e600-e11.
17. Facchinetti G, D'Angelo D, Piredda M, Petitti T, Matarese M, Oliveti A, et al. Continuity of care interventions for preventing hospital readmission of older people with chronic diseases: A meta-analysis. International Journal of Nursing Studies. 2020;101:103396. doi: 10.1016/j.ijnurstu.2019.103396
18. Ma C, McDonald MV, Feldman PH, Miner S, Jones S, Squires A. Continuity of Nursing Care in Home Health: Impact on Rehospitalization Among Older Adults With Dementia. Medical Care. 2021;59(10):913-20. doi: 10.1097/mlr.0000000000001599
19. Kalánková D, Kirwan M, Bartoníčková D, Cubelo F, Žiaková K, Kurucová R. Missed, rationed or unfinished nursing care: A scoping review of patient outcomes. Journal of Nursing Management. 2020;28(8):1783-97. doi: https://doi.org/10.1111/jonm.12978

For peer review only

20. Kalánková D, Stolt M, Scott PA, Papastavrou E, Suhonen R. Unmet care needs of older people: A scoping review. Nursing Ethics. 2021;28(2):149-78. doi: 10.1177/0969733020948112
21. Gustafsson N, Leino-Kilpi H, Prga I, Suhonen R, Stolt M, CA RcCA. Missed Care from the Patient's Perspective - A Scoping Review. Patient preference and adherence. 2020;14:383-400. doi: 10.2147/PPA.S238024
22. Hamel C, Garritty C, Hersi M, Butler C, Esmaeilisaraji L, Rice D, et al. Models of provider care in long-term care: A rapid scoping review. PLoS One. 2021;16(7):e0254527. doi: 10.1371/journal.pone.0254527
23. Schmüdderich K, Kiwitt J, Palm R, Roes M, Holle B. Core elements and potential of nurse-led care models in residential long-term care: A scoping review. Journal of Clinical Nursing. 2022;n/a(n/a). doi: https://doi.org/10.1111/jocn.16231
24. Arksey H, O'Malley L. Scoping studies: towards a methodological framework. International Journal of Social Research Methodology. 2005;8(1):19-32. doi: 10.1080/1364557032000119616
25. Bradbury-Jones C, Aveyard H, Herber OR, Isham L, Taylor J, O'Malley L. Scoping reviews: the PAGER framework for improving the quality of reporting. International Journal of Social Research Methodology. 2021:1-14. doi: 10.1080/13645579.2021.1899596
26. Daudt H, Mossel C, Scott S. Enhancing the Scoping Study Methodology: A Large, Interprofessional Team's Experience with Arksey and O'Malley's Framework. BMC Medical Research Methodology. 2013;13. doi: 10.1186/1471-2288-13-48
27. Peters MDJ, Marnie C, Tricco AC, Pollock D, Munn Z, Alexander L, et al. Updated methodological guidance for the conduct of scoping reviews. JBI Evidence Synthesis. 2020;18(10):2119-26. doi: 10.11124/jbies-20-00167
28. Levac D, Colquhoun H, O'Brien KK. Scoping studies: advancing the methodology. Implementation science. 2010;5(1):1-9. doi: 10.1186/1748-5908-5-69
29. Munn Z, Peters MDJ, Stern C, Tufanaru C, McArthur A, Aromataris E. Systematic review or scoping review? Guidance for authors when choosing between a systematic or scoping review approach. BMC Medical Research Methodology. 2018;18(1):143. doi: 10.1186/s12874-018-0611-x
30. Moher D, Shamseer L, Clarke M, Ghersi D, Liberati A, Petticrew M, et al. Preferred reporting items for systematic review and meta-analysis protocols (PRISMA-P) 2015 statement. Systematic Reviews. 2015;4(1):1. doi: 10.1186/2046-4053-4-1
31. Tricco AC, Lillie E, Zarin W, Colquhoun H, Levac D, Hempel S, et al. PRISMA Extension for Scoping Reviews (PRISMA-ScR): Checklist and Explanation. Annals of Internal Medicine. 2018;169(7):467-73. doi: 10.7326/M18-0850
32. National Institue on Aging. Enabling the future provision of long-term care in Canada. Toronto; 2019.
33. National Institute on Aging. What is long-term care? 2017 [Available from: https://www.nia.nih.gov/health/what-long-term-care.
34. Wysocki A, Butler M, Kane RL, Kane RA, Shippee T, Sainfort F. AHRQ Comparative Effectiveness Reviews. Long-Term Care for Older Adults: A Review of Home and Community-Based Services Versus Institutional Care. Rockville (MD): Agency for Healthcare Research and Quality (US); 2012.

For peer review only

35. World Health Organization. Continuity and coordination of care: a practice brief to support implementation of the WHO Framework on integrated people-centred health services. Geneva: World Health Organization; 2018 2018.
36. Chu CH, McGilton KS, Spilsbury K, Le KN, Boscart V, Backman A, et al. Strengthening International Research in Long-Term Care: Recommended Common Data Elements to Support Clinical Staff Training. Gerontology and Geriatric Medicine. 2021;7:2333721421999312. doi: 10.1177/2333721421999312
37. OECD. Health at a glance 2021: OECD Indicators. 2021
38. Davidson P, Halcomb E, Hickman L, Phillips J, Graham B. Beyond the rhetoric: what do we mean by a 'model of care'? Aust J Adv Nurs. 2006;23(3):47-55
39. Mheta D, Mashamba-Thompson TP. Barriers and facilitators of access to maternal services for women with disabilities: scoping review protocol. Syst Rev. 2017;6(1):99. doi: 10.1186/s13643-017-0494-7
40. Westerdahl F, Carlson E, Wennick A, Borglin G. Teaching strategies and outcome assessments targeting critical thinking in bachelor nursing students: a scoping review protocol. BMJ Open. 2020;10(1):e033214. doi: 10.1136/bmjopen-2019-033214
41. Bramer WM, de Jonge GB, Rethlefsen ML, Mast F, Kleijnen J. A systematic approach to searching: an efficient and complete method to develop literature searches. Journal of the Medical Library Association : JMLA. 2018;106(4):531-41. doi: 10.5195/jmla.2018.283
42. Booth A. Searching for qualitative research for inclusion in systematic reviews: a structured methodological review. Systematic Reviews. 2016;5(1):74. doi: 10.1186/s13643-016-0249-x
43. The EndNote Team. EndNote. 20 ed. Philadelphia, PA: Clarivate; 2022.
44. Ouzzani M, Hammady H, Fedorowicz Z, Elmagarmid A. Rayyan - a web and mobile app for systematic reviews. Systematic Reviews. 2016;2016(5):210. doi: 10.1186/s13643-016-0384-4
45. Polanin JR, Pigott TD, Espelage DL, Grotpeter JK. Best practice guidelines for abstract screening large-evidence systematic reviews and meta-analyses. Res Synth Methods. 2019;10(3):330-42. doi: 10.1002/jrsm.1354
46. Appelgren M, Bahtsevani C, Persson K, Borglin G. Nurses' experiences of caring for patients with intellectual developmental disorders: a systematic review using a meta-ethnographic approach. BMC Nursing. 2018;17(1):51. doi: 10.1186/s12912-018-0316-9
47. Page MJ, Moher D, Bossuyt PM, Boutron I, Hoffmann TC, Mulrow CD, et al. PRISMA 2020 explanation and elaboration: updated guidance and exemplars for reporting systematic reviews. BMJ. 2021;372:n160. doi: 10.1136/bmj.n160
48. Weingarten MA, Paul M, Leibovici L. Assessing ethics of trials in systematic reviews. BMJ. 2004;328(7446):1013-4. doi: 10.1136/bmj.328.7446.1013
49. Khalil H, Peters MD, Tricco AC, Pollock D, Alexander L, McInerney P, et al. Conducting high quality scoping reviews-challenges and solutions. J Clin Epidemiol. 2021;130:156-60. doi: 10.1016/j.jclinepi.2020.10.009
50. Pham MT, Rajić A, Greig JD, Sargeant JM, Papadopoulos A, McEwen SA. A scoping review of scoping reviews: advancing the approach and enhancing the consistency. Res Synth Methods. 2014;5(4):371-85. doi: 10.1002/jrsm.1123

For peer review only

51. Tricco AC, Lillie E, Zarin W, O'Brien K, Colquhoun H, Kastner M, et al. A scoping review on the conduct and reporting of scoping reviews. BMC Medical Research Methodology. 2016;16(1):15. doi: 10.1186/s12874-016-0116-4
52. Braun V, Clarke V. Thematic analysis - a practical guide. London: Sage; 2021.
53. Norwegian Ministry of Health and Care Services. ACT 2008-06-20 no. 44: Act on medical and health research (the Health Research Act) (2008).
54. Wager E, Wiffen PJ. Ethical issues in preparing and publishing systematic reviews. Journal of Evidence-Based Medicine. 2011;4(2):130-4. doi: https://doi.org/10.1111/j.1756-5391.2011.01122.x
55. ALLEA. The European Code of Conduct for Research Integrity. Berlin: Academy of Sciences and Humanities; 2017.
56. Ensuring Value in Research. Guiding principles Southampton: EVIR; 2022 [Available from: https://evir.org/our-principles/.
57. Skivington K, Matthews L, Simpson SA, Craig P, Baird J, Blazeby JM, et al. A new framework for developing and evaluating complex interventions: update of Medical Research Council guidance. BMJ. 2021;374:n2061. doi: 10.1136/bmj.n2061
58. Cullum N, Dumville J. Systematic reviews of the effects of interventions. In: Richards DA, Hallberg IR, editors. Complex interventions in health. Oxon: Routhledge; 2015.
59. Bradbury-Jones C, Aveyard H. The incomplete scope of scoping reviews: A framework for improving the quality of reporting. Journal of Clinical Nursing. 2021;30(21-22):e67-e8. doi: https://doi.org/10.1111/jocn.15998
60. Chalmers I, Glasziou P. Avoidable waste in the production and reporting of research evidence. The Lancet. 2009;374(9683):86-9. doi: 10.1016/S0140-6736(09)60329-9

FIGURE CAPTION

Box1. Operationalisation of core concepts
Table 1. PICOS framework
Table 2. Tentative data for extraction chart

For peer review only

For peer review only - http://bmjopen.bmj.com/site/about/guidelines.xhtml

PRISMA-P 2015 Checklist

This checklist has been adapted for use with systematic review protocol submissions to BioMed Central journals from Table 3 in Moher D et al: Preferred reporting items for systematic review and meta-analysis protocols (PRISMA-P) 2015 statement. *Systematic Reviews* 2015 **4**:1

An Editorial from the Editors-in-Chief of *Systematic Reviews* details why this checklist was adapted - Moher D, Stewart L & Shekelle P: Implementing PRISMA-P: recommendations for prospective authors. *Systematic Reviews* 2016 **5**:15

Section/topic	#	Checklist item	Information reported		Line number(s)
			Yes	No	
**ADMINISTRATIVE INFORMATION**					
**Title**					
Identification	1a	Identify the report as a protocol of a systematic review	x		1-2
Update	1b	If the protocol is for an update of a previous systematic review, identify as such		x	N/A
**Registration**	2	If registered, provide the name of the registry (e.g., PROSPERO) and registration number in the Abstract	x		56
**Authors**					
Contact	3a	Provide name, institutional affiliation, and e-mail address of all protocol authors; provide physical mailing address of corresponding author	x		4 – 22<br><br>Only email for corresponding author is provided.
Contributions	3b	Describe contributions of protocol authors and identify the guarantor of the review	x		305 - 309
**Amendments**	4	If the protocol represents an amendment of a previously completed or published protocol, identify as such and list changes; otherwise, state plan for documenting important protocol amendments		x	N/A
**Support**					
Sources	5a	Indicate sources of financial or other support for the review	x		311 - 313
Sponsor	5b	Provide name for the review funder and/or sponsor		x	N/A
Role of sponsor/funder	5c	Describe roles of funder(s), sponsor(s), and/or institution(s), if any, in developing the protocol		x	N/A

BioMed Central The Open Access Publisher

Section/topic	#	Checklist item	Information reported		Line number(s)
			Yes	No	
**INTRODUCTION**					
**Rationale**	6	Describe the rationale for the review in the context of what is already known	x		90 - 141
**Objectives**	7	Provide an explicit statement of the question(s) the review will address with reference to participants, interventions, comparators, and outcomes (PICO)	x		172 – 186 incl Table 1
**METHODS**					
**Eligibility criteria**	8	Specify the study characteristics (e.g., PICO, study design, setting, time frame) and report characteristics (e.g., years considered, language, publication status) to be used as criteria for eligibility for the review	x		188 – 204 incl Table 1 + Box 1
**Information sources**	9	Describe all intended information sources (e.g., electronic databases, contact with study authors, trial registers, or other grey literature sources) with planned dates of coverage	x		189
**Search strategy**	10	Present draft of search strategy to be used for at least one electronic database, including planned limits, such that it could be repeated	x		Supplemental material 2 +
STUDY RECORDS					
Data management	11a	Describe the mechanism(s) that will be used to manage records and data throughout the review	x		206 – 226
Selection process	11b	State the process that will be used for selecting studies (e.g., two independent reviewers) through each phase of the review (i.e., screening, eligibility, and inclusion in meta-analysis)	x		206 – 226
Data collection process	11c	Describe planned method of extracting data from reports (e.g., piloting forms, done independently, in duplicate), any processes for obtaining and confirming data from investigators	x		228 – 241
**Data items**	12	List and define all variables for which data will be sought (e.g., PICO items, funding sources), any pre-planned data assumptions and simplifications	x		Table 2
**Outcomes and prioritization**	13	List and define all outcomes for which data will be sought, including prioritization of main and additional outcomes, with rationale		x	N/A
**Risk of bias in individual studies**	14	Describe anticipated methods for assessing risk of bias of individual studies, including whether this will be done at the outcome or study level, or both; state how this information will be used in data synthesis		x	N/A
DATA					
**Synthesis**	15a	Describe criteria under which study data will be quantitatively synthesized	x		248 – 268

For peer review only

BioMed Central The Open Access Publisher

Section/topic	#	Checklist item	Information reported Yes	No	Line number(s)
	15b	If data are appropriate for quantitative synthesis, describe planned summary measures, methods of handling data, and methods of combining data from studies, including any planned exploration of consistency (e.g., I^2, Kendall's tau)		x	N/A
	15c	Describe any proposed additional analyses (e.g., sensitivity or subgroup analyses, meta-regression)		x	N/A
	15d	If quantitative synthesis is not appropriate, describe the type of summary planned	x		254 – 262 + table 2
**Meta-bias(es)**	16	Specify any planned assessment of meta-bias(es) (e.g., publication bias across studies, selective reporting within studies)		x	N/A
**Confidence in cumulative evidence**	17	Describe how the strength of the body of evidence will be assessed (e.g., GRADE)	x		236 - 238

For peer review only

BioMed Central
The Open Access Publisher

Additional file 2

Search blocks PubMed – tested 09.03.2023

Nr	Population - Older people	Mesh & All Fields
#1	Aged [MT]	3,438,107
#2	Elderly [MT]	3,438,107
#3	Older adult* [AF]	287,242
#4	Old [AF]	1,256,543
#5	Older people [AF]	432,325
#6	Senior* [AF]	91,963
#7	(((((#1) OR (#2)) OR (#3)) OR (#4)) OR (#5)) OR (#6)	4,706,859

Nr	Population - Nursing staff	Mesh & All Fields
#8	Allied health professional* [MT]	5,371
#9	Licenced practical nurse [MT]	936
#10	Nurse*[MT]	161,299
#11	Registered nurse* [AF]	21,175
#12	Licensed practice nurse* [AF]	3,094
#13	Registered practical nurse* [AF]	10,147
#14	Licensed vocational nurse* [AF]	338
#15	Nurse assistant* [AF]	16,897
#16	Health care assistant [AF]	72,382
#17	Formal caregiver [AF]	3,446
#18	Health care professional [AF]	811,662
#19	Practicing nurse* [AF]	125,708
#20	Professional nurs* [AF]	124,771
#21	Associate professional nurs* [AF]	22,698
#22	Nursing aide* [AF]	3,199
#23	Nursing assistant* [AF]	19,454
#24	Nurse specialist* [AF]	17,255
#25	((((((((((((#8) OR (#9) OR (#10) OR (#10)) OR (#11)) OR (#12)) OR (#13)) OR (#14) OR (#15)) OR (#16) OR (#17) OR (#18)) OR (#19)) OR (#20) OR (#21)) OR (#22) OR (#23)	977,683

Nr	Phenomenon of Interest - Continuity of care	Mesh & All Fields
#26	Continuity of patient care [MT]	285,774
#27	Continuity of care [AF]	297,752
#28	Continuity of nursing care [AF]	70,997
#29	Care continuity [AF]	297,752
#30	Informational continuity [AF]	135,277
#31	Relational continuity [AF]	257,556
#32	Interpersonal continuity [AF]	5,836
#33	Longitudinal continuity [AF]	28,001
#34	Management continuity [AF]	250,481
#35	((((((((((#26)) OR (#27)) OR (#28)) OR (#29)) OR (#30)) OR (#31) OR (#32)) OR (#33) OR (#34))	803,082

Nr	Phenomenon of Interest - Care & nursing	Mesh & All Fields
#36	Nursing care [MT]	141,040
#37	Nursing [MT]	262,763
#38	Patient* care need* [AF]	364,406
#39	Basic care [AF]	59,931
#40	Essence of care [AF]	2,524
#41	Essential care [AF]	89,592
#42	Fundamental care [AF]	22,791
#43	Eldercare [AF]	598
#44	Elderly care [AF]	78,561
#45	Geriatric care [AF]	71,595
#46	Care of older persons [AF]	122,045

For peer review only

Additional file 2

#47	Care of elderly persons [AF]	870,268
#48	Care of aged persons [AF]	888,440
#49	Old age assistance [AF]	2,261
#50	Care [AF]	3,134,474
#51	Care need* [AF]	592,274
#52	Physical care need* [AF]	66,455
#53	Basic nursing need* [AF]	4,453
#54	Physiological need* [AF]	75,595
#55	Biological need* [AF]	430,845
#56	Psychosocial need* [AF]	35,371
#57	Physical care [AF]	292,127
#58	Advanced care [AF]	182,347
#59	Support [AF]	11,411,024
#60	Relationship* [AF]	2,552,156
#61	Caring network [AF]	4,047
#62	Care plan [AF]	64,506
#63	Compassionate care [AF]	4,014
#64	Core nursing [AF]	10,215
#65	(((((((((((#36) OR (#37)) OR (#38) OR (#39) OR (#40) OR (#41) OR (#42)) OR (#43)) OR (#44)) OR (#45)) OR (#46) OR (#47)) OR (#48) OR (#49)) OR (#50) OR (#51) OR (#52)) OR (#53) OR (#54)) OR (#55) OR (#56) OR (#57) OR (#58)) OR (#59) OR (#60) OR (#61) OR (#62) OR (#63) OR (#64)	15,059,100

Nr	Phenomenon of Interest - Models, frameworks and interventions of care	Mesh & All Fields
#66	Nursing model [MT]	12,236
#67	Nursing process [MT]	86,531
#68	Critical pathway [MT]	7,710
#69	Clinical protocol [MT]	188,274
#70	Practice guideline [MT]	137,215
#71	Care model* [AF]	449,813
#72	Nursing framework* [AF]	24,500
#73	Care framework* [AF]	74,236
#74	Nursing approach [AF]	72,784
#75	Care approach [AF]	328,634
#76	Nursing programme [AF]	117,616
#77	Care programme [AF]	438,548
#78	Nursing intervention* [AF]	105,830
#79	Nurse-led [AF]	4,926
#80	Patient care pathway [AF]	27,988
#81	Patient pathway [AF]	260,075
#82	Care pathway [AF]	103,209
#83	Clinical pathway [AF]	208,344
#84	Clinical recommendation* [AF]	146,949
#85	Clinical path [AF]	87,189
#86	Clinical care plan [AF]	25,248
#87	Care path [AF]	9,242
#88	Care guideline [AF]	107,818
#89	Care recommendation* [AF]	113,660
#90	Care protocol [AF]	1,207
#91	((((((((((((((((((((((((((((#66)) OR (#67) OR (#68)) OR (#69) OR (#70) OR (#71)) OR (#72) OR (#73)) OR (#74) OR (#75) OR (#76) OR (#77) OR (#78)) OR (#79) OR (#80) OR (#81) OR (#82) OR (#83) OR (#84)) OR (#85) OR (#86) OR (#87) OR (#88) OR (#89) OR (#90)	2,061,156

Nr	Context - Long term care, home healthcare, home nursing, and other care facilities	Mesh & All Fields
#92	Community health services [MT]	329,298
#93	Home health services [MT]	125,852
#94	Home health nursing [MT]	375
#95	Long term care [MT]	28,240
#96	Primary health care [MT]	188,798
#97	Nursing home* [MT]	43,998
#98	Housing for the elderly [MT]	1,652
#99	Housing [MT]	36,444
#100	Primary care [AF]	570,684
#101	Community care [AF]	331,461

For peer review only

Additional file 2

#102	Community health services [AF]	438,391
#103	Municipal care [AF]	60,864
#104	Home care [AF]	961,037
#105	Home dwelling [AF]	30,403
#106	Home healthcare [AF]	74,095
#107	Home and community-based care [AF]	5,249
#108	Home and community-based services [AF]	4,519
#109	Longitudinal care [AF]	62,908
#110	Assisted living [AF]	30,390
#111	Independent living [AF]	37,878
#112	Residential care aged facilities [AF]	4,310
#113	Sheltered accommodation [AF]	309
#114	Sheltered housing [AF]	2,605
#115	Residential care [AF]	16,366
#116	Care home [AF]	161,305
#117	Day care [AF]	174,595
#118	Special accommodation [AF]	17,887
#119	((((((((((((((((((((((((((((#92)) OR (#93)) OR (#94) OR (#95)) OR (#96) OR (#97)) OR (#98) OR (#99) OR (#100)) OR (#101) OR (#102)) OR (#103) OR (#104) OR (#105) OR (#106) OR (#107)) OR (#108) OR (#109) OR (#110) OR (#111) OR (#112) OR (#113)) OR (#114) OR (#115) OR (#116)) OR (#117) OR (#118)	2,114,266

For peer review only
